# Men’s grief following pregnancy loss and neonatal loss: a systematic review and emerging theoretical model

**DOI:** 10.1186/s12884-019-2677-9

**Published:** 2020-01-10

**Authors:** Kate Louise Obst, Clemence Due, Melissa Oxlad, Philippa Middleton

**Affiliations:** 1 School of Psychology, University of Adelaide, Adelaide, South Australia Australia; 2 South Australian Health and Medical Research Institute, Adelaide, South Australia Australia

**Keywords:** Men, Fathers, Grief, Stillbirth, Miscarriage, Neonatal loss, Systematic review

## Abstract

**Background:**

Emotional distress following pregnancy loss and neonatal loss is common, with enduring grief occurring for many parents. However, little is known about men’s grief, since the majority of existing literature and subsequent bereavement care guidelines have focused on women. To develop a comprehensive understanding of men’s grief, this systematic review sought to summarise and appraise the literature focusing on men’s grief following pregnancy loss and neonatal loss.

**Methods:**

A systematic review was undertaken with searches completed across four databases (PubMed, PsycINFO, Embase, and CINAHL). These were guided by two research questions: 1) what are men’s experiences of grief following pregnancy/neonatal loss; and 2) what are the predictors of men’s grief following pregnancy/neonatal loss? Eligible articles were qualitative, quantitative or mixed methods empirical studies including primary data on men’s grief, published between 1998 and October 2018. Eligibility for loss type included miscarriage or stillbirth (by any definition), termination of pregnancy for nonviable foetal anomaly, and neonatal death up to 28 days after a live birth.

**Results:**

A final sample of 46 articles were identified, including 26 qualitative, 19 quantitative, and one mixed methods paper. Findings indicate that men’s grief experiences are highly varied, and current grief measures may not capture all of the complexities of grief for men. Qualitative studies identified that in comparison to women, men may face different challenges including expectations to support female partners, and a lack of social recognition for their grief and subsequent needs. Men may face double-disenfranchised grief in relation to the pregnancy/neonatal loss experience.

**Conclusion:**

There is a need to increase the accessibility of support services for men following pregnancy/neonatal loss, and to provide recognition and validation of their experiences of grief. Cohort studies are required among varied groups of bereaved men to confirm grief-predictor relationships, and to refine an emerging socio-ecological model of men’s grief.

**Trials registration:**

PROSPERO registration number: CRD42018103981

## Background

The loss of a pregnancy through miscarriage or stillbirth, and the death of a baby within the first 28 days of life, are typically unexpected and highly distressing events for parents. In addition to processes of grief and bereavement, both pregnancy loss and neonatal loss can be complicated due to the additional loss of hopes for raising a child, and potential ambiguity regarding status as a parent [1–4]. Grief following both forms of loss can be described as disenfranchised [5]. This is due to a lack of social recognition for the unborn baby as a living individual, along with an absence of cultural norms and understanding about how to mourn the death of a baby [2, 6]. Societal norms may minimise the loss, particularly in the case of miscarriage [7].

### Background and context

Global estimates indicate that miscarriage occurs for approximately one in four recognised pregnancies, while every year, 2.6 million babies worldwide are stillborn, and a further 2.8 million die within the first week of life [8–11]. The majority of these losses occur in low and middle income countries [11]. However, pregnancy/neonatal loss also remains a significant health burden in high income countries, where despite advances in medical technologies, rates of stillbirth have remained stagnant for over two decades [12–14].

Definitions of pregnancy loss according to gestational age vary considerably across countries, with over 30 different stillbirth classification systems identified across the literature [10, 15]. The World Health Organization (WHO) recommends a definition of stillbirth as a loss after 28 weeks’ gestation, whereas in the United Kingdom (UK) a stillbirth is classified after 24 weeks, and in the United States of America (USA), Canada, and Australia, after 20 weeks [16–20]. Losses prior to these gestations are considered a miscarriage. Despite this variability, there is currently limited evidence to suggest that grief following pregnancy loss is affected by gestational age [3, 21–25].

### Previous literature on grief following pregnancy loss and neonatal loss

Growing recognition of the impact of pregnancy/neonatal loss has led to increased research interest into the psychological and emotional burden on bereaved parents and families [26–28]. There is widespread consensus that grief is a multifaceted and highly individual process, although there may be general similarities. For example, early models of grief described common ‘stages’ of grief, from shock or denial through to acceptance or recovery [29, 30]. The Dual Process Model of Coping with Bereavement [31] described an ongoing oscillation between ‘loss-orientated’ (emotional) and ‘restoration-oriented’ (problem-solving) coping strategies. Specific to bereaved parents, the continuing bonds approach recognises the need for ongoing connections through symbolic objects, rituals, and sharing memories [32, 33]. Finally, research on gender and grief has found that due to social expectations surrounding how men should behave, men are generally less likely to outwardly display emotional reactions. Men may also experience more difficulty than women in seeking or accepting help for mental health concerns, grief, and adjustment to loss [34–36].

Following pregnancy/neonatal loss, men engage more frequently than women in compensatory behaviours (such as increased substance use), score higher on avoidance scales, and experience difficulty in approaching or accessing support services [37–41]. Despite these difficulties, the majority of previous research and subsequent pregnancy/neonatal loss bereavement care guidelines have focused primarily on the experiences and needs of heterosexual mothers [42–45]. Fewer studies and recommendations relate to men’s experiences of grief and subsequent support needs. Given the potential for detrimental health and wellbeing outcomes among men following pregnancy/neonatal loss, it is essential to further understand how men grieve, and the factors that contribute to worsened or improved outcomes [21, 46, 47]. Recently, three reviews were published in areas relating to men’s experiences of pregnancy/neonatal loss. However, two of these were scoping reviews rather than systematic [48, 49], and the other thematically synthesised only qualitative studies on men’s lived experiences of miscarriage [50]. This systematic review aimed to provide a comprehensive summary and appraisal of existing qualitative and quantitative literature on men’s grief, following both pregnancy loss and neonatal loss. The study objectives were to identify (1) how men experience grief following pregnancy loss and neonatal loss, and (2) the factors and/or predictors that contribute to men’s grief.

## Methods

### Data sources and search strategy

Following the Preferred Reporting Items for Systematic Reviews and Meta-Analyses (PRISMA) guidelines [51], a systematic literature search of four online databases (PubMed, PsycINFO, Embase, and CINAHL) was completed in October 2018. Initially, preliminary searches were undertaken across the databases to identify potential subject headings and keywords. Following this, the final search strategies were developed in collaboration with an experienced research librarian (see Additional file 1: for search strategies).

### Study selection

Inclusion criteria were qualitative, quantitative, or mixed methods studies, published between 1998 and October 2018, reporting the results of primary data on men’s grief and/or predictors of grief following pregnancy loss or neonatal loss. By definition, this included the death of a baby at any stage in-utero, or up to 28 days after a live birth. Exclusion criteria were articles not published in English, abstracts, editorials or opinion pieces, discussion or review articles not reporting primary data, and studies using a comparator (e.g., women) that did not present the data pertaining to men separately. Studies were also excluded if they investigated the grief experiences of men who had experienced an elective abortion or termination of pregnancy for viable foetal anomaly, as there is literature to suggest that these types of losses may lead to different psychological outcomes compared to other forms of pregnancy loss [52, 53].

### Study yield

**Fig. 1:**
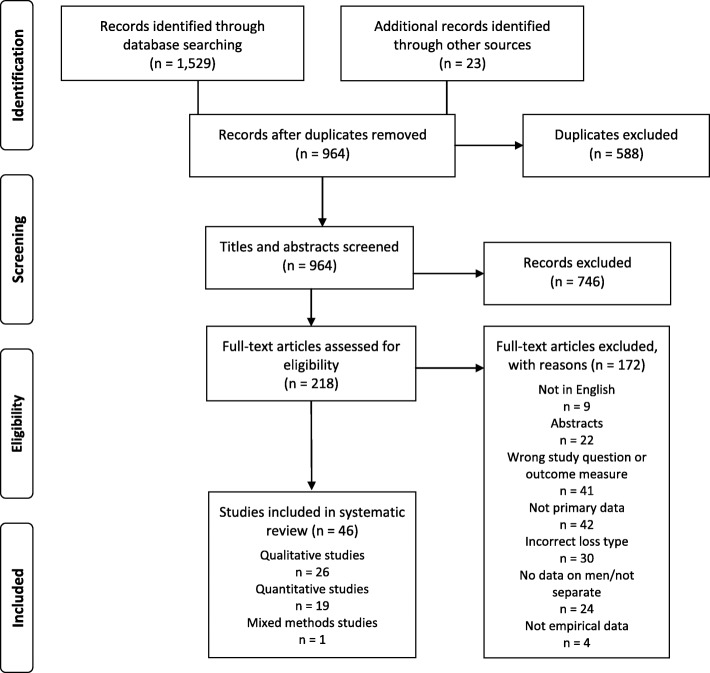
PRISMA Flow Diagram

The database searches identified 1529 potentially eligible studies. A further 23 articles were sourced manually from database-identified articles and systematic reviews [27, 37, 38], resulting in a total of 1552 articles. Following removal of duplicates and screening, a total of 46 studies were selected for inclusion in the final analysis and were agreed upon by all authors (See Fig. 1 for the PRISMA flow diagram). A random subset of 10% of potentially eligible studies was co-screened by all authors. Interrater agreement was high (*K* = .72–.96, *p* < .05) with any discrepancies resolved by consensus discussion.

### Data extraction and study appraisal

The findings of the included articles were extracted by the first author using a predesigned data extraction form. The second author then cross-checked this information. The table items included research setting/country, date of publication, study design, number and characteristics of participants, key findings on men’s grief experiences, measures of grief, and/or predictors of grief. Study quality and risk of bias were assessed using the Critical Appraisal Skills Program (CASP) quality appraisal checklists [54] for qualitative studies, cohort studies, and randomised control trials (RCTs) where appropriate. The first author reviewed and rated all of the included studies, and the second author cross-checked a random sample (5%) of the same studies. Discrepancies between the reviewers were resolved through group discussions.

## Results

### Description of studies

#### Design

Nineteen papers were quantitative, 26 qualitative, and one used a mixed methods design [55]. For ease of discussing results in this paper, the mixed methods study was classified as qualitative, as the emphasis of reporting was clearly on this form of data. Thirty-nine studies were peer-reviewed papers, and seven were unpublished theses [56–62]. All but one of the included quantitative studies were variations of cohort designs, most commonly using structured questionnaires to assess grief. The remaining study was a RCT, examining the effectiveness of nurse-care and self-care interventions on grief following miscarriage [63]. Qualitative studies predominantly used individual semi or unstructured interviews. However, two studies used a postal [55] or online questionnaire [64], one used focus groups [65], and one was an autoethnography [66]. Details of each of the 46 studies can be found in Additional file 2. Table 1 provides an overview of studies by research design.

**Table 1:** Overview of included studies

	Quantitative (*n* = 19)	Qualitative (*n* = 26)	Mixed Methods (*n* = 1)	Total (*n* = 46)
Year of publication				
1998–2002	7	5	–	12
2003–2007	5	6	–	11
2008–2012	2	7	–	9
2013–2018	5	8	1	14
Region of study				
Australia	5	5	–	10
United Kingdom	3	3	–	6
United States and Canada	8	13	1	22
Europe	3	4	–	7
The Middle East	–	1	–	1
Informant group				
Men	3	13	–	16
Men and women	15	12	1	28
Men and service providers	–	1	–	1
Men, women and service providers	–	1	–	1
Total study sample size^a^				
10 or under	1	9	–	10
11–50	3	16	–	19
51–100	3	–	1	4
101–200	6	1	–	7
201–300	2	–	–	2
301–500	2	–	–	2
500+	2	–	–	2
Number of male participants				
10 or under	1	18	–	19
11–50	6	7	1	14
51–100	4	–	–	4
101–200	4	1	–	5
201–300	–	–	–	–
301–500	3	–	–	3
Unspecified	1	–	–	1
Loss type				
Miscarriage	9	8	–	17
Recurrent miscarriage (3+)	1	1	–	2
Stillbirth	1	8	1	10
Neonatal death	–	–	–	–
Medical termination for nonviable anomaly	1	1	–	2
Combination (pregnancy and neonatal losses)	7	8	–	15
Primary outcome focus				
Grief	16	5	1	22
Other	3	21	–	24

^*a*^Numbers only report the number of participants who experienced a pregnancy loss or neonatal loss

#### Focus

Twenty-one studies investigated grief experiences following miscarriage (definitions which ranged between ≤20–24 weeks’ gestation), 10 following stillbirth, and 15 following a combination of loss types. Two papers explored experiences following termination of pregnancy for nonviable (or lethal) foetal anomalies [67, 68]. No papers focused exclusively on neonatal death. Twenty-three studies (16 quantitative and seven qualitative) focused on grief as a primary outcome. The remaining included elements of grief secondary to general explorations of experiences of loss, including ‘meaning’ [69], ‘impact’ [46] and ‘emotional responses’ [70] among others [23, 57–59, 64, 65, 68, 71–83]. Two qualitative [73, 74] and two quantitative [24, 84] studies also investigated grief following pregnancy loss that continued into a subsequent pregnancy or after the birth of a child.

### Participant characteristics

Twenty-two studies were based in the USA and Canada, 10 in Australia, six in the UK, and seven in Europe (six Swedish). One study was based in the Middle East [69], one interviewed African-American couples [77], and another two interviewed Australian couples who were born in the Middle East [71, 72]. The majority of participants across remaining studies were Caucasian, with those including mixed ethnicities providing little to no discussion on cultural or ethnic background. All studies were conducted in high-income countries, and male participants were heterosexual men who experienced pregnancy loss with a female partner. With the exception of six studies that did not specify men’s marital status [64, 65, 79, 80, 85, 86], the majority of male participants were in a relationship with the partner they were with at the time of loss. Sixteen studies recruited only men [25, 46, 57, 58, 60, 61, 64, 66, 70, 75, 80–83, 85, 87]. The remaining studies included men as participants in conjunction with their female partner.

Samples sizes varied widely, from one (an autoethnography) [66] to 131 men [64] in qualitative studies, and nine [70] to 341 men [23] in quantitative studies (see Additional file 2**:** for details). Thirteen studies reported age and standard deviations (*SD*s) for male participants [23–25, 56, 60, 61, 73, 84, 87–91]. Across these, the average age of 1052 men was 33 years (pooled *SD* = 8.74). The remaining studies either did not report male participant ages [64–66, 68, 71, 72, 78, 79, 82, 92], combined men’s ages with women’s [63, 69, 74, 76, 86, 93], or provided an average age and/or range [46, 55, 57–59, 62, 67, 70, 75, 77, 80, 81, 83, 85, 94–96]. The youngest participant was aged 20 years [70], and the oldest 61 years [57] at the time of study participation.

### Quality of included studies

An assessment of quality was undertaken for each study using CASP checklists [54]. Study quality varied, however the overall standard was acceptable and therefore no studies were excluded based on poor quality. With the exception of 12 studies, [25, 55, 68, 81, 86, 88–90, 92, 94–96], almost all studies used convenience, purposive or snowball sampling to recruit participants. While ethically justified given the sensitive nature of the research, the results may therefore not be representative of all men bereaved to pregnancy/neonatal loss more broadly. This is further indicated by the narrow range of variability in participant characteristics. All studies adhered to appropriate ethical standards including obtaining informed consent, protecting participant anonymity through identification numbers or pseudonyms, and offering contact details of pregnancy/neonatal loss support services to bereaved parents in case of distress. However, 10 studies did not state whether institutional ethical approval had been sought or obtained [25, 46, 79, 82, 83, 86, 87, 91, 93, 94]. Two studies also acknowledged potential conflicts of interest relating to the first author being the developer of the intervention under investigation [63], and another employed by the bereavement service being evaluated [92]. Otherwise, no additional conflicts were declared by study authors or identified as a result of quality rating.

Qualitative studies were generally of a high standard, with methodologies and analyses (content [46, 55, 60, 64, 69], thematic [61, 65, 71, 72], grounded theory [59, 62, 68], autoethnographic [66], descriptive [83] and phenomenological [56–58, 73–75, 77–82]) clearly reported and justified in the context of ‘exploratory’ or ‘understanding lived experience’ research aims. Quantitative studies reported either correlational and regression analyses [24, 67, 76, 86, 88, 90, 92, 93], or group difference tests [23, 25, 76, 84, 87, 89, 91, 94–96], including significance testing of resulting relationships or differences. However, one small quantitative study reported only numbers and percentages of participants who endorsed a particular feeling relating to grief or service outcome [70], and another reported percentages of participants who had received certain support services following a loss [95].

With the exception of one study which employed author-developed measures of grief and support service satisfaction [70] the remaining quantitative studies employed standardised and validated measures for both predictors and grief [23–25, 67, 76, 84, 86–96]. However, there was an inconsistency in the use of grief measures and reporting grief. Although 13 studies used the Perinatal Grief Scale (PGS) as a primary measure of grief, some reported average total grief scores [67, 88, 90, 93, 95], others average subscale scores [84, 96], both [25, 87, 91], or subscale correlations to predictor variables [24, 89]. Finally, 17 studies also grouped together different types of loss as part of the investigation of grief (e.g., miscarriage and stillbirth, stillbirth and neonatal death, or all three types together) [24, 46, 57–61, 73, 77, 80, 83, 84, 86, 88–90, 93]. As a result, outcomes specific to these different loss groups may have gone unrecognised. Only two studies specifically discussed differences in support and grief between miscarriage and stillbirth [46, 61].

### Findings relating to the grief experience

#### Quantitative studies

Thirteen quantitative studies used the PGS as the primary measure of grief [24, 25, 67, 84, 87–93, 95, 96]. Other grief measures included the Grief Experience Inventory-Loss Version (GEI-L) [94], the Revised Impact of Miscarriage Scale (RIMS) [23, 76, 96], the Miscarriage Grief Inventory [63], and the Texas Revised Inventory of Grief (TRIG-F) [86]. Although primarily a measure of stress rather than grief, three studies also used the Impact of Events Scale (IES) [67, 91, 94].

Of the 12 quantitative studies that provided raw grief scores for men, outcomes varied considerably both between studies and within them [23, 25, 67, 76, 84, 87, 88, 91, 93–96]. This was indicated by wide range and *SD*s. Average total grief scores for men in studies using the PGS varied from 36 [93] to 133.19 [95] from a possible range of 33 to 165. However, the majority of average total PGS scores across remaining studies were between 73 and 83, with *SD*s ranging between values of 16 and 22 [25, 87, 88, 91, 93]. Population norms suggest that total grief scores above 91 for the PGS are reflective of a high degree of grief [97]. The outcomes reported across studies here (with the exception of one study [96]) indicate that men typically are not scoring in the highly significant grief range. However, they are nevertheless scoring quite highly in general [25, 67, 84, 87, 88, 91, 93] (see Table 2 for a comparison of studies reporting total *M* and *SD* scores for the PGS). Similarly, for three studies using the RIMS as a measure of grief, outcomes also varied with subscale scores ranging from 0 to 57 [96], 5 to 24 [76], and subscale *SD*s up to 4.08 [23].

**Table 2:** Comparison of total grief scores on the Perinatal Grief Scale

Scale	Study	Loss type	Time point (*n*)	Mean (*SD*)	Overall classification (degree of grief based on normative data)^^^
Perinatal Grief Scale (total scores)	Barr (2004)	Stillbirth (≥ 20 weeks gestation) or neonatal death (≤ 28 days from birth)	1 month post-loss (*n* = 72)	82.7 (20.73)	Mid
			13 months post-loss (*n* = 69)	71.9 (24.57)	Low
	Conway & Russell (2000)	Miscarriage (losses occurred between 5 and 16 weeks of gestation)	Within 3 weeks of loss (*n* = 32)	133.19 (18.98)	High
			2–4 months post-loss (*n* = 16)	136.31 (24.11)	High
	Franche & Bulow (1999)	Perinatal loss (losses occurred between 10 to 42 weeks of gestation)	Pregnant subsequent to loss group: 1–31.5 months post-loss (*n* = 24)	74.66^a^ (7.16^a^)	Low
			Loss group (not currently pregnant): 2–19 months post-loss (*n* = 18)	75.11^a^ (5.8^a^)	Low
	Johnson & Puddifoot (1998)	Miscarriage (< 24 weeks of gestation)	Within 11 weeks post-loss (*M* = 5.5 weeks; *n* = 158)	78.4 (22.7)	Mid
	Puddifoot & Johnson (1999)	Miscarriage (≤ 20 weeks of gestation) or stillbirth (> 20 weeks of gestation)	NR (*n* = 323)	80.98 (29.08)	Mid
	Rich (2000)	Ectopic pregnancy, miscarriage or stillbirth (losses occurred between 3 and 42 weeks of gestation)	2–60 months post-loss (*M* = 16.5 months; *n* = 114)	73.99 (18.47)	Low
	Serrano & Lima (2006)	Miscarriage (≤ 24 weeks of gestation)	Up to 1 year post-loss (*n* = 30)	72.23 (16.85)	Low
	Volgsten et al. (2018)	Miscarriage (up to 21 + 6 weeks of gestation)	1 week post-loss (*n* = 64)	44.5^a^ (*SD*s NR)	Low
			4 months post-loss (*n* = 64)	37.5^a^ (*SD*s NR)	Low
	Wilson et al. (2015)^#^	Stillbirth (from at least 20 weeks of gestation or over 400 g in weight)	6–8 weeks post-loss (*n* = 9)	82.8^a^ (7.31^a^)	Mid
			6 months post-loss (*n* = 6)	75.9^a^ (7.02^a^)	Low
			13 months post-loss (*n* = 3)	63.9^a^ (5.80^a^)	Low

^a^Calculated based on reported subscale mean and *SD* scores; ^^^normative data as reported in Lasker & Toedter (2000); ^#^grief reported for fathers who held their stillborn baby after birth; *NR* not reported

This variation in grief scores may be due to inconsistencies in the timing of grief measurement. Time since the loss varied from one week in one study [96], to 32 years in another [67]. Overall, it was not clear whether increased time since the loss led to reduced grief in men (see Table 2). However, some studies also noted that even when the losses had occurred many years in the past, participants’ grief had not necessarily diminished with time [55, 67, 79, 83].

In nine of 10 studies which compared men and women, men’s grief scores were significantly lower or less intense than those of women [67, 94, 96]. This was indicated by approximately 20 points of difference on the PGS and IES [88, 91, 93], and 3 points of difference on the RIMS [23, 76]. Importantly, however, some studies noted that the use of existing grief measures (including the PGS and RIMS) might not be valid for measuring men’s grief experiences, particularly in relation to potential differences between internal versus external grieving styles [23, 84, 89, 95]. There were mixed findings in terms of overall scale scores across similar studies looking at grief following miscarriage, with Despair (internalised grief) scores higher in men than those for Active Grief (externalised grief) in two studies [87, 95], and lower in the remaining two [91, 96]. Across other grief measures, men scored highly on the Devastating Event (RIMS), Denial and Social Desirability (GEI-L), and Avoidance (IES) subscales [23, 76, 91, 94, 96]. This may represent some of the more inward responses to loss involved in some men’s grief experiences.

#### Qualitative studies

In 14 qualitative studies, men reported that the loss of their baby was a significant life event, regardless of gestational or neonatal age [46, 57–62, 66, 73, 75, 79, 81, 82, 85]. However, other men in 10 studies (some overlapping with the above 14 studies) also reported less intense reactions, including stating that their partners experienced worse grief in comparison to them [56, 61, 69, 71–75, 78, 79]. Regardless of grief intensity, in 14 studies men seemed to face additional or unique tasks and challenges that complicated their experience, or delayed the timing of grief. These included a sense of helplessness or powerlessness (especially during labour) [66, 69, 75, 79, 81], and responsibilities such as caring for other children, completing paperwork, organising a funeral/burial, and informing family and friends [46, 57, 58, 61, 62, 66, 80–82].

Although the grief experience was highly varied, and subsequent grieving styles mixed, there was a general trend among male participants towards instrumental grieving, which included the use of active or problem-focused coping strategies [55–62, 65, 66, 70–75, 77–82]. ‘Keeping busy’ and ‘moving forward’ were common desires [55, 59, 73, 77, 78, 80], with men seeking out distractions including sporting activities or increased exercise [58, 59, 62], returning to work [57–61, 72, 74, 79, 80], completing household tasks [58, 61, 73, 81], and creative, hands-on outlets such as woodworking, painting or writing [57, 58, 66]. However, men in 10 studies also reported outward emotional grief expressions such as crying. Although, these were frequently kept private, with many men preferring to grieve independently and alone [46, 56–59, 62, 66, 81, 82, 85].

### Findings relating to predictors of men’s grief

Of the included quantitative studies, 16 included an analysis on predictors of men’s grief and/or correlations to related factors [23–25, 67, 76, 84, 86–93, 95, 96]. As part of a wider exploration of grief, all qualitative studies also discussed factors that contributed (both positively and negatively) to men’s grief. Overall, a wide range of varied predictors/factors were considered, which fell broadly into four domains or levels: (1) individual/person-level factors; (2) interpersonal factors; (3) community/sociocultural factors; and (4) public policy factors.

### Individual factors

#### Attachment to the baby

One of the strongest factors found to impact upon grief at the individual level was men’s attachment to the baby. In 11 qualitative studies, men who had developed a bond with their baby throughout the pregnancy described more intense grief following a subsequent loss [46, 58, 60–62, 73, 75, 79, 81, 82, 85]. However, in five studies some men stated that they did not feel that they had a relationship with the developing baby [61, 69, 75, 79], either because it was an early miscarriage, or they described little involvement during the pregnancy. Others also made a conscious attempt during pregnancy not to get attached, due to previous experience of loss or diagnosis of a life-threatening condition [74]. In these cases, grief was reported as less intense. Actions that increased attachment included spending time with the baby [66, 85], and attending ultrasound appointments to ‘see’ the baby and hear the heartbeat [46, 61, 62, 66, 73, 79, 82, 85]. Although estimates of grief were imprecise due to a small male sample size, one quantitative study measuring grief after seeing or holding the stillborn baby identified worsened grief for men [92]. Similarly, men in six qualitative studies who held or spent time with their baby following a stillbirth generally also reported high levels of grief [58, 62, 66, 77, 81, 85]. Importantly, however, the cause and effect relationship here is unclear. It may be that men who spent time with their baby were already more attached, and therefore more likely to experience worsened grief.

Seven quantitative studies explored men’s attachment to the developing baby using measures including viewing an ultrasound [25], vividness of visual imagery [87], increasing gestational age [23–25, 67, 93], and holding or seeing the baby following stillbirth [92]. Men who viewed an ultrasound image had an average PGS total score 23 points higher than those who did not view any images [25], and men with a strong visual image of their baby as measured by the Baby Vividness of Visual Imagery Questionnaire (“vivid imagers”) had an average PGS total score 40 points higher than those who did not [87]. Again, the causal relationship here is unclear.

Attachment may be related to gestational age, since a longer pregnancy could result in more opportunities for bonding. In five quantitative studies, increasing gestational age was associated with higher grief scores [23–25, 67, 93]. However, qualitative studies complicated this picture. In studies inclusive of multiple loss types, men who had experienced earlier losses did not describe less intense grief than those with later losses [46, 57–59, 61]. Studies on miscarriage also noted that men’s grief responses were not dissimilar to the grief of men described in studies focused on stillbirth or neonatal death [62, 75, 82]. As such, the impact of gestational age on grief remains unclear.

#### Men’s personality

Two studies on the same sample of bereaved parents in Australia [88, 90] investigated the relationships between grief and a general personality proneness to guilt (considering one’s actions as regretful) and shame (attributing regretful actions to oneself). Overall, shame and guilt-proneness were found to explain 63% of the variance in grief (as measured by the PGS) in men, with shame-proneness accounting for 56% of the variance in men’s grief 13 months following a stillbirth or neonatal death [88]. In the follow-up study [90], which conducted analysis within the couple, women’s self-conscious emotions and grief tendencies did not appear to influence men’s emotions and grief tendencies (although men’s did impact upon women’s). Franche [24] similarly explored the predictive value of self-criticism on grief after pregnancy/neonatal loss. Considered in combination with other obstetric and demographic variables, higher levels of self-criticism were significantly associated with higher scores on all subscales of the PGS in men (*p* < .01 for the Active Grief subscale, and *p* < .001 for Despair and Difficulty Coping subscales).

#### Demographic factors

Findings relating to the relationship between demographic factors and grief were mixed. Only one quantitative study [23] found age to be a significant predictor of grief following miscarriage, with men aged < 35 years scoring higher on the Devastating Event subscale of the RMIS. The remaining quantitative studies including age as a predictor did not find a significant association [24, 93, 95], and qualitative studies did not specifically explore or discuss the impact of age on grief. However, the majority of men who participated in qualitative studies were generally aged 28 years or over, with the exception of two studies which reported minimum ages of 20 and 21 years [46, 77].

Ethnicity did not emerge as a significant predictor of grief, but this was rarely explored. One study comparing Swedish and American couples’ experiences of miscarriage [76] found differences between the samples on one subscale of the RMIS (Loss of Baby). However, this difference was attributed to linguistic understanding and wording of the scale questions, rather than the grief experience itself. Other quantitative studies including a small number of culturally diverse participants (e.g., African American, Asian-Australian, Hispanic, Native American) either did not examine differences [23, 88–90, 93], or did not find any significant differences in grief [91]. Five qualitative studies had mixed ethnic samples (e.g., Jamaican, African-American, Hispanic/Latino), but none reported any differences in grief; although, their aim was not to do so [57, 60, 62, 73, 82]. Further, in two Australian-based studies of the same sample of participants with Middle-Eastern backgrounds, culture was not discussed as impacting upon grief [71, 72]. In one qualitative study based in Israel [69], high drop-out rates were noted due to (mostly) the husband’s objection to participating, in the context of a typically “closed” religious society. Finally, in a study of low-income African-American parents, grief for men did not differ to those in other studies. However, “dealing with stressful life events”, including economic hardship and other unrelated family deaths, were found to compound grief for both parents [77].

In one quantitative study [67], involvement in organised religious activity was inversely associated with Despair subscale scores on the PGS for men (*p* = 0.047). In eight qualitative studies, men who reported religious or spiritual beliefs also found this to be a source of comfort in coping with their grief. This was both from a meaning-making perspective (e.g., “what God does, He does it for the best”) [69], and from the additional social support that was received from religious/church communities [58, 59, 62, 73, 77, 81, 82]. However, the experience of loss for some men in two qualitative studies also led to questioning or challenging of their religious beliefs [66, 69].

#### Recurrent loss and living children

Findings relating to the impact of previous losses and number of living children on grief were also varied. In one quantitative study which examined men who had experienced recurrent miscarriage, grief and stress scores were high on both the PGS (*M* = 72.23, *SD* = 16.85), and IES (*M* = 26.53, *SD* = 13.76) [91]. In contrast, men with a history of loss in nine qualitative studies [46, 61, 62, 68, 71, 72, 78, 82, 83] did not report different or increased levels of grief. Yet, in four studies, men did report increased worry about future pregnancies [59, 62, 75, 78].

In two quantitative studies including subsequent pregnancy status as an indicator of grief intensity, no significant relationships were found between a group who were currently pregnant following a loss, and a group who had not had a subsequent pregnancy or child [84, 89]. However, in three qualitative studies examining experiences of grief into subsequent pregnancies/children, it was clear that men’s grief did continue, along with added concerns and vigilance due to the knowledge of potential risks [73, 74, 80]. Similarly, one of three studies examining the presence of living children at the time of loss found a relationship to worsened grief in men [23]. However, for the remaining two studies including this factor, it was unrelated [86, 89]. Four qualitative studies described how living children could both enhance the reality of the developing baby (thus worsening grief), *and* make coming to terms with the loss easier. This was attributed to enhanced appreciation for surviving children, reassurance about the possibility of successful future pregnancies, or providing a caring role to focus on [58, 75, 78, 81].

### Interpersonal factors

#### Quality of the partner relationship

In 10 qualitative studies, men noted that the relationship with their partner could be either a positive or negative contributor to the grief experience [55, 57, 59–62, 70, 71, 74, 81]. For many participants, a lack of recognition for their grief from family, friends and healthcare professionals meant their partner became their main source of interpersonal support [59, 61, 81]. Although many men reported supportive relationships with “frank and honest communication” [81] resulting in a stronger couple bond that buffered the grief experience, many also experienced conflict or relationship strain due to incongruent grieving styles [55, 57, 59, 61, 62, 70, 74, 75, 81]. Where dissonant grieving styles or conflict were present, men reported a sense of alienation or frustration that added to their grief experience [55, 60, 61, 74]. However, despite early conflict, where couples learned to effectively navigate one another’s grief, the relationship was ultimately strengthened [59, 62, 74].

#### The supporter role

Although not a factor quantified for measurement in any quantitative studies, one of the most consistently reported and important elements relating to men’s grief across qualitative studies was being a ‘supporter’ to their female partner and family. Twenty-three qualitative studies identified an element of the supporter role from men’s responses [46, 55–59, 61, 62, 65, 68, 69, 71–75, 77–82, 85]. In 21 of these, all male participants reported their primary role of being the supporter to their female partner. In the remaining two, the majority of men (five of nine [62], and 14 of 15 [75]) also reported this role. For men in five studies, the need to support their partner explicitly came from a perception that she had a more intense grief reaction in comparison to themselves [59, 61, 69, 74, 79]. In 15 studies, men described having to suppress or put aside their own grief to take on this role [46, 57–59, 61, 62, 68, 71, 72, 74, 75, 77, 81, 82, 85]. As a result, many of these men reported a feeling of being ignored or unrecognised as grievers, instead seen merely as the ‘support person’ [46, 61, 80]. In three studies, some men reported feeling as though this supporter role was helpful, as it gave them a meaningful task to focus on [69, 73, 75]. However, for other men in Hamama Raz et al. [69] and the remaining studies, this role ultimately served as a hindrance in allowing them to acknowledge, express and manage their grief and emotional responses [46, 56–59, 61, 62, 65, 68, 69, 71, 72, 74, 75, 79–82, 85].

#### Support and acknowledgement from family and friends

In 16 studies looking at support, 10 found family and/or friends to be a helpful facilitator to men’s coping following the loss [56, 57, 59, 66, 72, 73, 75, 79, 81, 82]. This was important, since many men explicitly reported a preference not to engage in formal counselling [78] and/or support groups [56, 61]. However, men’s experiences of support from family and friends varied greatly. In the one quantitative study that looked at family and friend support as variables, ‘talking with friends’ was associated with increased grief scores, along with ‘timing of talking to family’. However, there is no description of what is meant by this [93]. In the remaining qualitative studies, the majority of men also reported talking with either close family members or friends post-loss, which they found meaningful and helpful most of the time [56, 57, 59, 61, 75, 79, 81, 82]. Practical support immediately following the loss (e.g., making meals) was particularly appreciated by men in three qualitative studies [61, 72, 82]. For others “subtle” gestures of care from other male friends, including sharing their own stories or scheduling time/activities post-loss, were immense comforts [66, 81, 82]. However, seven qualitative studies also reported negative – or a total absence of – interactions with family and friends [59–62, 73, 75, 80]. In two of these studies, men did not feel the need to discuss their grief with anyone other than their partners, or avoided talking to others about the loss, believing this would reduce the impact [73, 75]. In the remaining five, men desired support from family and friends, however stated that “no one” [80] was available to them due to a lack of understanding, avoidance, and/or discomfort [59–62]. Where there was a lack of acknowledgement or support from family and friends, reported grief experiences were worsened [60, 61, 80].

#### Support and acknowledgement from healthcare professionals

Similar to support from family and friends, the role of healthcare professionals was recognised in one quantitative study [70] and 13 qualitative studies [46, 56, 60–62, 64, 65, 68, 72, 73, 78, 81, 82] as essential to the bereavement process. However, among studies that examined healthcare provider support, findings were again mixed. In 10 studies, some men reported positive experiences with healthcare staff [46, 60, 61, 64, 68, 73, 75, 78, 81, 82]. Three studies noted that providers who worked “extra hard” to provide both medical and practical information to men were valued [81], and parents who experienced the support of specialist bereavement care teams, or follow-up telephone calls from care providers, commented positively on this [68, 78]. However, men in one quantitative study felt excluded from services and none were satisfied with the support they received from health professionals [70]. Likewise, other men in 11 of both the same and different qualitative studies also reported negative interactions with healthcare staff. This led to sadness, anger, or distress which worsened or prevented the grieving process [46, 61, 62, 64, 65, 70, 72, 75, 79, 81, 82]. Common issues included insensitive language or confusing medical terminology [79, 81, 82], a lack of answers or explanations [61, 62], a lack of practical information on how they could care for their female partner or organise a funeral/burial [46, 62, 72], and failing to recognise their distress and role as a father [46, 64, 65, 70, 75, 79]. It should be noted that the majority of studies reporting negative experiences with health care providers/the hospital focused on miscarriages as opposed to later-term losses, with the exception of three which focused exclusively on stillbirth [64, 65, 81]. Two studies exploring healthcare support following both miscarriage and stillbirth also noted differences in care between these types of losses, with miscarriages receiving considerably less support in comparison to stillbirths [46, 61].

### Community factors

#### Disenfranchisement of grief following pregnancy/neonatal loss

A lack of community acknowledgement and understanding for grief following pregnancy loss was explicitly identified by male participants in seven qualitative studies from the USA [58, 65, 66, 82], Ireland [46, 78] and Australia [61]. Across these, men discussed widespread taboo, stigma and silence surrounding miscarriage and/or stillbirth which worsened their grief. Experiences of disenfranchisement included questioning their identity as fathers due to confusion surrounding whether their pregnancy was understood as a baby or not [46], only discussing their loss if/when prompted by another bereaved parent [78], and hurtful comments from others which minimised their grief or encouraged them to “move on” from the loss [61, 82]. Overall, this sense of disenfranchisement due to a lack of community acknowledgement for pregnancy loss led men to experience increased distress and feelings of isolation [46, 58, 61, 66, 82]. This factor was not explored in quantitative studies.

#### Male role expectations and attitudes toward men’s grief

Tying in closely with the ‘supporter role’ theme, a pressure to conform to masculine role expectations toward how men should grieve was expressed in 19 qualitative studies. These were based in Australia [61, 72, 85], the UK [79], the USA [55–60, 62, 65, 66, 74, 80, 82], Ireland [46], Sweden [81] and Israel [69]. No quantitative studies explored this factor. In 13 studies, male participants specifically discussed the need to be “strong”, and a perceived expectation to hide their grief [46, 56–59, 61, 62, 72, 74, 79, 80, 82, 85]. Men reported that these expectations had a direct negative impact on their grieving process, as they felt prevented from displaying their emotions in front of others, seeking support, and/or working through their grief [46, 57, 61, 65, 66, 74, 80, 82, 85]. This expectation to hide their emotions also meant that the impact of the loss on these men was frequently disguised from family, friends and healthcare professionals. This led to a generalised lack of recognition for their grief, and a further sense of disenfranchisement, above that which already exists for grief following pregnancy/neonatal loss generally [60, 61, 82].

### Public policy factors

#### Woman-focused maternity care and support services

A general focus on woman-centred care in the hospital environment and existing support services was identified as a factor impacting grief by nine qualitative studies, but not in quantitative studies [46, 60, 61, 64, 66, 70, 80–82]. A general community attitude that pregnancy and subsequent loss was primarily a “woman’s experience” [80] was explicitly expressed by men in three studies [46, 60, 80]. Men also reported feeling overlooked or ignored in the context of existing healthcare and support services. For example, in the hospital environment, both following loss and during subsequent pregnancies, men felt “out of place” [81], “marginalised” [46] and sometimes, as though they “barely existed” [61]. Similar sentiments were echoed in the context of support services/groups which were delivered primarily by women and focused on “‘traditionally feminine’ modes of grieving” [60, 61, 66, 80]. Men in five studies expressed a desire for recognition [80–82], as well as a need for increased male involvement in care and support services [46, 61]. Indeed, in studies where male friends and family were available to men, or healthcare staff sought to specifically involve them in pregnancy care and support services, grief improved [46, 60, 61, 64, 66].

#### Workplace policies: bereavement leave

Another consistent theme at a policy level was the availability of paternity or bereavement leave for men following pregnancy/neonatal loss. Returning to work following loss was explicitly discussed in 11 qualitative studies [57–62, 66, 74, 79, 80, 82] and one quantitative study [86]. For the majority of men, particularly those who described a more instrumental grieving style, work provided a distraction from their loss, and was used as a strategy to cope with their grief [57–60, 74, 79]. However, four qualitative studies, which examined men’s experience of returning to work in more depth, identified varied outcomes [61, 66, 80, 82]. In three of these studies, men were not provided with the same opportunities as their female partners to take paid leave from work following their loss [61, 66, 80]. This led to physical and emotional exhaustion, along with difficulties in concentration and keeping up with tasks. In one quantitative study [86], men also reported difficulty returning to work. In contrast, the burden of grief was eased for men in two studies who were offered extended paid leave or extensions on work-related deadlines [61, 82].

### The emerging model: a socio-ecological theory of men’s grief

**Fig. 2:**
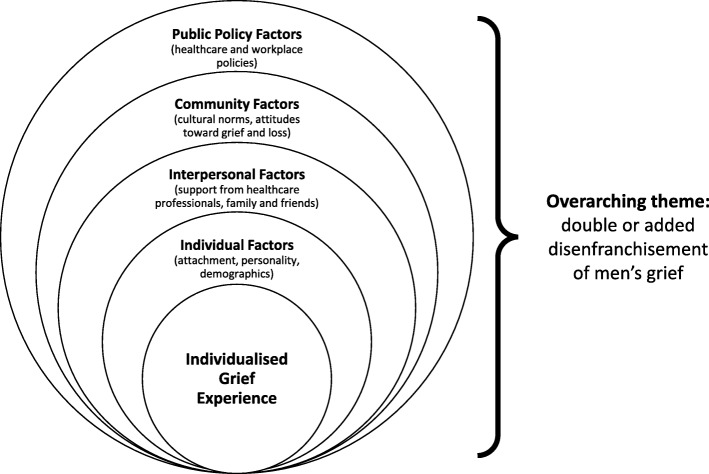
Socio-Ecological Model of Men's Grief

Spanning the individual, interpersonal, community and public policy realms, the factors identified in this review align with a socio-ecological approach to understanding grief. We propose a preliminary model of men’s grief, adapted from Bronfenbrenner’s [98] Ecological Systems Theory (see Fig. 2). The original theory (focusing more broadly on development as opposed to grief) purported that an individual’s development is impacted by four interacting levels in the environment: the microsystem (the immediate environment), the mesosystem (settings in which we actively participate), the exosystem (wider social setting), and the macrosystem (culture and belief systems) [98]. Like the original theory, the model of men’s grief proposed here acknowledges that the grief experience does not exist in isolation. Rather, it is shaped by a complex system of interacting factors and levels. These include those relating to the individual, their relationships, the surrounding community, and governing policies. Each of these levels also interacts with one another in a bi-directional nature. For example, cultural norms and beliefs regarding men’s roles – particularly in pregnancy – may play a vital role in informing the woman-centred focus of perinatal healthcare and bereavement leave policies (and vice versa). These norms can also impact the ways that individuals interact with one another in response to pregnancy/neonatal loss, as do these interpersonal interactions serve to support the overarching cultural norms. At the centre, the individual, their personality, knowledge, attitudes, and skills are impacted by, and continually interact with, all of these contributors.

The overarching theme of this model is the concept of “double disenfranchisement”, first introduced by Cacciatore and Raffo [99] in their study on lesbian maternal bereavement. The authors argued that given an additional lack of societal recognition for their status as legitimate mothers, lesbian women can experience an added level of disenfranchisement following pregnancy loss [99]. In a similar way, the lack of recognition that many men cited in the included papers for their position as grieving fathers indicates that they may also experience a sense of added or double disenfranchisement. Consequently, it is imperative that men’s grief following pregnancy/neonatal loss is not viewed entirely as an individual response to the event, but as part of a wider socio-ecological process.

## Discussion

### Main findings and implications

This systematic review has summarised men’s experiences of grief following pregnancy/neonatal loss, and identified factors that contribute towards grief. Evidence from this synthesis and the proposed socio-ecological model of grief highlights potential ways to support men, including access to multi-level strategies.

### Grief

Both quantitative and qualitative studies revealed the highly varied and individual nature of men’s grief. Although men’s grief was less intense compared to women’s in some quantitative studies [23, 67, 76, 88, 91, 93, 94, 96], qualitative studies identified the significant impact of loss on men. Given grief is a normal and expected process following a loss, it is unsurprising that some men experienced such significant effects. In contrast to stereotypes that men intellectualise or rationalise their grief, studies also found that men do grieve on an emotional level. They may also oscillate between problem-focused coping and emotional expressions of grief, as reflected in the dual-process model of coping [31]. However, men’s experiences also appeared to be consistent with the theory of disenfranchised grief [5], with a general silence surrounding pregnancy loss contributing to feelings of isolation and worsened grief. Compared to women overall, men may also face different challenges that can worsen grief. This finding is consistent with previous research on gender and grieving which suggests that grief can be impacted by, but is not dependent on, gender [100].

### Predictors of grief

A wide range of factors have the potential to influence men’s grief. At an individual level there are mixed findings relating to demographic factors, suggesting that these have not been well-explored. Similarly, personality constructs may play a key role in predicting grief [24, 88, 90], although further research is required to confirm causality. However, in contrast to early assumptions that men only develop an attachment to the developing baby as gestation increases, results suggest that attachment at any level is an important predictor of grief [58, 62, 79, 81, 82].

Men’s interactions with others seem to play a pivotal role in how they experience grief. The quality of the couple relationship contributed to either a positive source of support that helped the grief process, or a negative source of added stress which increased the impact of the loss [55, 57, 59–62, 70, 71, 74, 81]. Grief was eased when friends and family were available to support men, and were understanding of their loss [56, 57, 59, 61, 75, 79, 81, 82]. Furthermore, a positive experience with the healthcare system led to both reduced grief and increased support group participation [60], whereas insensitive treatment led to psychological distress and worsened grief [64].

These findings relating to individual and interpersonal factors are similar to studies focused on women’s experiences of grief following pregnancy/neonatal loss. For example, findings on demographic factors have also been inconsistent. Involvement in religious activity and strength of religious faith have been inversely associated with grief in some studies [101, 102] but not others [103]. Similarly, maternal age both has [23, 101], and has not [24, 104], been found to be a significant predictor of grief. However, while the impact of cultural diversity is yet to be explored in men, cross-cultural studies with women highlight a range of culturally-specific understandings and practices relating to the loss of a baby that can impact upon grief [105–109]. Finally, social support and experiences with the healthcare system have been linked to both immediate grief and long-term psychological health for women [1, 110–113].

Alongside the potential for mixed styles of grieving, individual-level supports should consider these factors to provide tailored and appropriate support options to suit men’s individual needs. For example, individual counselling or support groups may not be appealing to all men. Rather, previous research has recommended creative options including activity-based supports, evidence-based online supports, opportunity for peer contact, or including male support workers in hospitals [21, 46, 47]. Joint couple bereavement counselling could also be considered where necessary. As a minimum, it is important to provide explanations to bereaved parents about incongruent grieving between partners, and skills to navigate potential issues. There is an ongoing need for healthcare professionals to provide sensitive and empathetic care to both members of a couple relationship. This includes adopting appropriate, jargon-free language, providing explanations relating to the cause of loss when available, and follow-up calls specifically to men in the weeks or months following a loss. Practical information on how best to support their partner, alongside recognising and managing their own grief, was also desired by men [46, 62, 72].

Community attitudes concerning the legitimacy of parents’ grief following pregnancy/neonatal loss, along with gendered expectations relating to how men should behave in the face of loss, are important in shaping men’s experience. A lack of recognition for grief following pregnancy/neonatal loss resulted in disenfranchisement [5], with men frequently reporting a feeling of being overlooked as grieving fathers [46, 58, 61, 66, 82]. Policies relating to woman-centred care and bereavement leave in the workplace also impacted grief. Where pregnancy was seen as an issue relating exclusively to women, and men consequently felt excluded from the loss experience at the hospital, their grief was worsened [46, 61, 81]. A small number of studies also suggested that men were frequently not afforded adequate workplace leave to manage their grief following a loss [61, 66, 80]. In line with recent investigations which have highlighted similar social and economic consequences of stillbirth [27, 114], there is potential to re-examine current paternity and bereavement leave policies [66, 80].

These findings imply that beyond individual and interpersonal supports, there is also a need to educate the community about the impact of pregnancy/neonatal loss on men, as well as promoting their strengths to seek and accept, rather than avoid, support. More generally, similar recommendations have been made in the men’s physical and mental health literature, where stigma surrounding male help-seeking frequently serves as a barrier to accessing appropriate health-related supports [34–36]. Strategies are also needed to develop male-inclusive healthcare practices, and promote the meaningful engagement of men as equal partners throughout pregnancy and childbirth. In the broader postnatal health context, engagement of fathers has demonstrated improved long-term physical and mental health outcomes for women, men and babies [115, 116].

### Limitations and future research

Although inconsistencies concerning grief between quantitative and qualitative studies highlight the varied nature of men’s experiences, some authors have questioned the ecological validity of current grief measures [23, 84, 89, 95]. The PGS, for example, was initially developed and validated in a sample of mainly bereaved mothers (women *n* = 138 and men *n* = 56) [117]. As such, some of the items and subscales have been criticised for measuring more traditionally ‘feminine’ (or intuitive) expressions of grief, which may under-recognise more ‘masculine’ (or instrumental) expressions and responses. Across included studies that provided separate subscale analyses of grief, the greatest differences between men and women occurred on the Active Grief subscale. This reflects outward expressions of grief and emotions, which men often display less frequently than women [25, 91, 95, 96], and may indicate a selection bias in qualitative studies toward men with more extreme grief responses. However, some men in qualitative studies also expressed less extreme reactions to the loss, indicating representation of a range of experiences [56, 61, 69, 71–75, 78, 79]. Given the correlational nature of findings on viewing an ultrasound [25], it also remains unclear as to whether viewing an ultrasound results in more intense grief, or whether men who were already more attached to their baby were more likely to attend the ultrasound appointment. This concept requires further investigation.

Overall, quantitative studies seem to have captured part of the picture about grief, focusing predominately on individual and interpersonal factors as key contributors to the grief experience. Further studies are needed to explore the unique facets of men’s grief following pregnancy/neonatal loss (e.g., helplessness, marginalisation, and the expectation to ‘be strong’), as well as the broader sociocultural and public policy factors. This might include a more comprehensive measure of attachment to the baby and workplace functioning, or quantitative measures of marginalisation from the healthcare system, and the expectation to ‘be strong’ and conform to masculine norms. Once these factors are well understood, there will be scope to develop and validate a grief measure with increased sensitivity toward these elements, as well as the more instrumental-orientated grief styles [23, 80, 84].

None of the included studies focused exclusively on men’s grief following neonatal loss. Furthermore, those which did include men experiencing neonatal loss did not specifically identify disenfranchisement as a contributing factor. This may be due to increased recognition for the baby’s life, given survival outside of the womb. However, in studies on neonatal loss not eligible for inclusion [4, 118, 119], parents reported feelings similar to those following miscarriage or stillbirth. These included loneliness and isolation from friends and family, as well as a profound “silence concerning the death” [4]. There is a need for updated research to explore men’s experiences of grief following neonatal loss, and to identify any unique factors impacting grief.

Finally, participants in the included studies were predominately Caucasian, heterosexual males. As ever, there is a need for research among diverse samples of men. This includes gay and transgender men whose pregnancy and loss experiences may involve unique or added challenges [120, 121], single and separated men who experience relationship breakdown following a loss, and culturally and socio-demographically diverse men. The emerging socio-ecological model of men’s grief following pregnancy/neonatal loss also requires refinement and confirmation through cohort studies which includes these diverse populations. A comprehensive longitudinal study following men throughout pregnancy, and then during and following a pregnancy/neonatal loss, would also be useful to explore the causal pathways for risk and protective factors of grief.

## Conclusions

A socio-ecological model of men’s grief implies a need for multi-level strategies, rather than individual bereavement supports alone. Tailored support is needed for instrumental grievers, and to address the unique challenges men face. Additional strategies may also include community campaigns to change attitudes toward grief and loss and promote the strengths, rather than weaknesses, of traditionally normative “masculine” traits including resilience and strength to seek assistance. Appropriate workplace policies and health systems that validate and engage men throughout pregnancy, childbirth, and in the event of loss, are also required. A focus on men’s grief and subsequent support does not seek to reduce the significance of the loss for their female partners. Rather, a lack of validation as equal partners in the pregnancy and loss process has led to increased difficulties in coping for men, and being afforded acknowledgement for their grief [82, 85]. As such, this review provides a helpful synthesis on the existing literature for men’s grief following pregnancy/neonatal loss, and a solid theoretical foundation from which future research and recommendations can be built.

## Supplementary information

**Additional file 1:** Search strategies by database

**Additional file 2:** Overview of studies

## Data Availability

All data is contained within the manuscript file and its additional files.

## Abbreviations

CASP: Critical Appraisal Skills Program
GEI-L: Grief Experience Inventory-Loss Version
IES: Impact of Events Scale
PGS: Perinatal Grief Scale
PRISMA: Preferred Reporting Items for Systematic Reviews and Meta-Analyses
RCT: Randomised Control Trial
RIMS: Revised Impact of Miscarriage Scale
SD: Standard Deviation
TRIG-F: Texas Revised Inventory of Grief
UK: United Kingdom
USA: United States of America
WHO: World Health Organization
