# Supplementary material for: Men’s grief following pregnancy loss and neonatal loss: a systematic review and emerging theoretical model
Source: BMC Pregnancy Childbirth. 2020 Jan 10;20:11. doi: 10.1186/s12884-019-2677-9 (PMC6953275; doi:10.1186/s12884-019-2677-9)
Supplement: Supplementary file 1 — Additional file 1: Search strategies by database [file 12884_2019_2677_MOESM1_ESM.docx]

**Appendix 1.** Search strategies by database

PubMed

| **Men** | **Grief** | **Pregnancy/child loss** |
| --- | --- | --- |
| “men”[mh] men[tiab] “male”[mh] male[tiab] males[tiab] “fathers”[mh] father*[tiab] “spouses”[mh] spouse*[tiab] partner*[tiab] husband*[tiab] paternal*[tiab] paternity[tiab] masculinity[mh] masculin*[tiab] | “bereavement”[mh] bereave*[tiab] grief*[tiab] griev*[tiab] mourn*[tiab] “attitude to death”[mh] | “abortion, spontaneous”[mh] spontaneous abortion*[tiab] “stillbirth”[mh] stillbirth*[tiab] still birth*[tiab] stillborn*[tiab] “still born*”[tiab] “pregnancy, ectopic”[mh] ectopic pregnanc*[tiab] “fetal death”[mh] fetal death*[tiab] foetal death*[tiab] fetus death*[tiab]  foetus death*[tiab] pregnancy loss*[tiab] miscarriage*[tiab] “perinatal death”[mh] perinatal death*[tiab] “perinatal mortality”[mh] perinatal mortalit*[tiab] “infant mortality”[mh] infant mortalit*[tiab] “infant death”[mh] infant death*[tiab] infant loss*[tiab] neonatal mortal*[tiab] neonatal death*[tiab] neonatal loss*[tiab] baby's death*[tiab]  baby death*[tiab] |

PsycINFO

| **Men** | **Grief** | **Pregnancy/child loss** |
| --- | --- | --- |
| human males.sh men.ti,ab male.ti,ab males.ti,ab fathers.sh father$.ti,ab spouses.sh spouse$.ti,ab partner$.ti,ab  husband$.ti,ab paternal$.ti,ab paternity.ti,ab masculinity.sh masculin$.ti,ab | bereavement.sh bereave$.ti,ab grief.sh grief$.ti,ab griev$.ti,ab mourn$.ti,ab | spontaneous abortion.sh spontaneous abortion$.ti,ab stillbirth$.ti,ab still birth$.ti,ab stillborn$.ti,ab still born$.ti,ab ectopic pregnanc$.ti,ab fetal death$.ti,ab foetal death$.ti,ab (fetus adj4 death$).ti,ab (foetus adj4 death$).ti,ab pregnancy loss$.ti,ab miscarriage$.ti,ab perinatal death$.ti,ab perinatal mortalit$.ti,ab infant mortalit$.ti,ab infant death$.ti,ab infant loss$.ti,ab neonatal mortal$.ti,ab neonatal death$.ti,ab neonatal loss$.ti,ab baby$ death$.ti,ab |

Embase

| **Men** | **Grief** | **Pregnancy/child loss** |
| --- | --- | --- |
| ‘male’/de male:ti,ab males:ti,ab men:ti,ab father/de father*:ti,ab spouse/exp spouse*:ti,ab partner*:ti,ab  husband*:ti,ab ‘paternal behavior’/de paternal*:ti,ab paternity:ti,ab ‘masculinity’/de masculin*:ti,ab | bereavement/de bereave*:ti,ab grief/exp grief*:ti,ab griev*:ti,ab mourn*:ti,ab ‘attitude to death’/de | ‘spontaneous abortion’/de ‘spontaneous abortion*’:ti,ab miscarriage*:ti,ab ‘ectopic pregnancy’/de ‘ectopic pregnanc*’:ti,ab ‘fetus death’/de ‘fetal death*’:ti,ab ‘foetal death*’:ti,ab (fetus NEXT/4 death*):ti,ab (foetus NEXT/4 death*):ti,ab stillbirth/de stillbirth*:ti,ab ‘still birth*’:ti,ab  stillborn*:ti,ab ‘still born*’:ti,ab ‘pregnancy loss*’:ti,ab ‘perinatal death’/de  ‘perinatal death*’:ti,ab ‘perinatal mortality’/exp ‘perinatal mortalit*’:ti,ab ‘infant mortalit*’:ti,ab ‘infant death*’:ti,ab ‘infant loss*’:ti,ab ‘newborn mortality’/de ‘newborn mortalit*’:ti,ab ‘neonatal mortal*’:ti,ab ‘neonatal death*’:ti,ab ‘neonatal loss*’:ti,ab ‘baby* death*’:ti,ab ‘death of a baby’:ti,ab |

CINAHL

| **Men** | **Grief** | **Pregnancy/child loss** |
| --- | --- | --- |
| MH men TI men AB men MH male TI male* AB male* MH men’s health TI “men’s health” AB “men’s health” MH fathers TI father* AB father* MH expectant fathers MH spouses TI spouse* AB spouse* TI husband* AB husband* MH paternal behavior TI paternal* AB paternal* TI paternity AB paternity MH masculinity TI masculin* AB masculin* | MH bereavement+ TI bereave* AB bereave* TI grief* TI griev* AB grief* AB grief* TI mourn*  AB mourn* MH attitude to death | MH abortion, spontaneous TI “spontaneous abortion*” AB “spontaneous abortion*” TI miscarriage* AB miscarriage* MH pregnancy, ectopic TI “ectopic pregnanc*” AB “ectopic pregnanc*” MH perinatal death TI “perinatal death*” AB “perinatal death*” TI stillbirth* AB stillbirth* TI “still birth*” AB “still birth*”  TI stillborn* AB stillborn* TI “still born*” AB “still born*” TI “pregnancy loss*” AB “pregnancy loss*” TI “fetal death*” AB “fetal death*” TI “foetal death*” AB “foetal death*” TI (fetus n4 death*)  AB (fetus n4 death*)  TI (foetus n4 death*)  AB (foetus n4 death*) MH infant mortality TI “infant mortalit*” AB “infant mortalit*” TI “infant loss*” AB “infant loss*” TI “perinatal mortalit*” AB “perinatal mortalit*” TI “neonatal mortalit*” AB “neonatal mortalit*” TI “neonatal death*” AB “neonatal death*” TI “neonatal loss*” AB “neonatal loss*” TI “baby death*” AB “baby death*” TI “baby’s death*” AB “baby’s death*” |
