# Supplementary material for: Men’s grief following pregnancy loss and neonatal loss: a systematic review and emerging theoretical model
Source: BMC Pregnancy Childbirth. 2020 Jan 10;20:11. doi: 10.1186/s12884-019-2677-9 (PMC6953275; doi:10.1186/s12884-019-2677-9)
Supplement: Supplementary file 2 — Additional file 2: Overview of studies [file 12884_2019_2677_MOESM2_ESM.docx]

**Appendix 2.** Overview of studies

Qualitative Studies

| Author (Year) | Study Aims (objectives; focus) | Participants/Setting (*N*; loss type; country; time since loss) | Method/Design (recruitment; data collection; type of analysis) | Grief Experience (key themes relating to the grief experience) | Key Predictors of Grief (factors impacting the grief experience) |
| --- | --- | --- | --- | --- | --- |
| Abboud & Liamputtong (2005) | To investigate coping strategies, social support and satisfaction with health care among ethnic women and their partners following miscarriage | Six women and their male partners from Melbourne, Australia with miscarriage experience; ethnic backgrounds (Middle East); all Christian; four couples with living children | Recruited via snowball sampling; one couple through GP referral; data collected using in-depth interviews with women and men separately; used thematic analysis | Men hid their feelings to stay strong for their partners: “I kept myself strong but was hurting inside”. Men tried not to make the miscarriage a big issue in their lives, instead coping by trying to return to ‘normal’. | Men felt that their role during miscarriage was one of support and encouragement. Men felt that support from family and friends was helpful for female partners but not themselves. Many would have liked information from the hospital about what to expect and how to care for their partner. |
| Abboud & Liamputtong (2003) | To examine the experience of miscarriage for women and their partners, providing an account of what happened before, after, and during the time of their miscarriage. | Six women and their male partners from Melbourne, Australia with miscarriage experience; ethnic backgrounds (Middle East); all Christian; four couples with living children | Recruited via snowball sampling; one couple through GP referral; data collected using in-depth interviews with women and men separately; data analysed using thematic analysis | Men had to consider their partner first and hold back their emotions in order to be in control of the situation. Most men reported feeling happy now and thought little about the miscarriage. | Men’s role during the time of miscarriage was to support their partner. Some did not have much discussion about the miscarriage with their partner. Subsequent children made the miscarriage easier to come to terms with. |
| Armstrong (2001) | To explore fathers’ experiences of pregnancy after a prior perinatal loss. | Four U.S. couples experiencing pregnancy after prior perinatal loss; prior losses had occurred between 12 and 20 weeks gestation; three men were Caucasian and one was of Jamaican descent | Recruited through treating healthcare providers at clinics and medical practices; data collection using two unstructured interviews – the follow-up interview was 3-4 weeks after the first to validate and further examine emerging concepts | Men reported that the intensity of the loss was greater than anticipated. Those who were more invested in the pregnancy experienced greater grief; those who were less attached were more prepared to “move on, forget about it”. Despite differing responses at the time of loss, all described similar emotions during the current pregnancy, which were attributed to the previous loss. | Death rituals (holding baby, memorial service) bought closure to fathers. Activities and “keeping busy” assisted with managing grief. All men described a need to protect their partner, however none expressed displeasure with the supporter role, as it was helpful to have a caring responsibility. All described positive experiences with support from HCPs and family/friends. Three felt belief in God was a comfort during grief. |
| Bonnette & Broom (2012) | To explore men’s experiences of stillbirth and how they experienced fathering and grief. | 12 men bereaved to stillbirth from NSW, Australia. Men were aged between 28 and 54; for two the stillbirth was their first child; one was a twin birth | A combination of purposive and snowball sampling. Data were collected using in-depth interviews (45 mins-2.5 hours); data analysed using interpretive phenomenological analysis | Masculine cultural scripts regarding emotional expression were evident; tension emerged between ‘manly’ considerations and men’s realities of negotiating and expressing grief. There was a cultural expectation that they remain strong and stoical. Men experienced an initial inexpressiveness of emotion to ‘protect’ their female partners. | Each man connected or bonded with their unborn baby (especially through ultrasound attendance/viewing). Time with the baby post-birth allowed men to reconcile the stillbirth experience with their fathering identity. Tension arose between wanting recognition as a male and recognition as a grieving father. Overall, being recognized and validated as a grieving father, not merely as a supportive partner, was important. |
| Brierly (2018) | To explore couples’ experiences grieving late-term pregnancy loss on Facebook. | Nine couples bereaved by stillbirth (26-41 weeks gestation) in the U.S.; all couples were Caucasian and in married heterosexual relationships; time since loss ranged from 3-12 months | Recruited via purposive sampling through online pregnancy loss organisations/groups and local women’s health centres; data were collected using semi-structured interviews and analysed using interpretive phenomenological analysis | Grief responses varied: some men were openly emotional, whereas others spoke to their role as the husband/man, who needed to “be the strong one” to protect and comfort his wife. Many felt their grief navigation was more internal than their wives’: they were more information-seeking and task-oriented. Many discussed their desire to grieve privately, or with one close friend or family member. Several expressed guilt or failure that they were unable to protect their wife and unborn child from harm. A few associated emotions with weakness, however more encouraged other males experiencing stillbirth to express themselves. | The hospital experience played an important role in the bereavement process. Although women were more likely than men to use Facebook for grieving, all participants talked about using Facebook to navigate grief. The “memories” function and targeted advertising were particularly harmful to the grief process, as these served as a constant reminder that they no longer needed baby supplies or parenting resources. |
| Cacciatore (2013) | To evaluate fathers’ experiences of stillbirth and psychosocial care. | 131 fathers who had experienced a stillbirth (> 22 weeks gestation) between 2000 and April 2010 in Sweden. Majority of losses occurred two years prior to assessment; 95% of fathers were present at the birth. | Recruited via purposive sampling through the Swedish National Infant Foundation website. Data collected using an online questionnaire (82 items covering demographic info and stillbirth topics); analysed qualitatively using inductive manifest content analysis. | 16% of fathers felt sadness, anger or being hurt: dominantly this related to their fatherhood being unrecognised or invalidated. | 86% of fathers expressed gratitude for compassionate and professional provider care. Men were thankful when HCPs legitimised their fatherhood by treating the baby as if s/he was born alive. Insensitive treatment was the primary reason fathers perceived transgressions and subsequent psychological distress/grief. |
| Campbell-Jackson et al. (2014) | To explore mothers’ and fathers' experiences of becoming a parent to a child born after a recent stillbirth, covering the period of the second pregnancy and up to two years after the birth of the next baby. | Seven couples with British and Polish backgrounds who had experienced stillbirth (>24 weeks gestation). Subsequent children were an average of 16.6 months old (SD = 7.4 months). | Mothers who had taken part in a previous study on stillbirth were approached, along with their partners, via an invitation letter. Data were collected using semi-structured individual interviews with women and men separately, and analysed using interpretive phenomenological analysis. | Although all fathers described worry and fear throughout subsequent pregnancy, some thought that their experience was less intense than their wives’. Fathers described managing their own anxieties whilst focussing on practical tasks to provide support for their spouse. | Some fathers found it difficult to find space to grieve as they felt they needed to stay strong in order to support their spouse. Being at a different stage of grief from their spouse was also highlighted as a challenge. For fathers, work was the most reported source of distraction. |
| Cholette (2012) | To enhance understanding of the paternal experience of perinatal loss, to help inform future care and support to bereaved families. | Seven fathers who had experienced perinatal loss (death of a fetus in-utero or neonatal death up to the first 28 days of life) in the U.S. or Canada. Ages ranged from 34-61 years; all men were married and Christian. Time since loss ranged from 3 months to 28 years. | Purposive sampling through obstetrical health care providers. Data were collected using individual interviews, and analysed using Hermeneutic interpretive phenomenology | Regardless of the type, all reported a deep sense of lasting loss which changed their lives. Some fathers began to work diligently on home or work projects as a form of time out. All of the men expressed concern for their spouse, and this took precedence over their own grief and wellbeing. Although healing transpired with time, the loss was never forgotten. | Fathers expressed a need to be a protective father/husband/man by demonstrating strength and stoicism. As a result, the paternal grief was commonly left unacknowledged not only by society but by themselves. Communication and support from family and friends were crucial to men’s ability to cope. |
| Colon (2009) | To explore paternal grief experiences following pregnancy loss, to gain knowledge of the grief process and perceptions of fathers. | Nine fathers who had experienced perinatal loss (from conception until 28 days after birth) in the U.S. Men were aged between 41 and 78 years, time since loss ranged from 7 to 54 years. All had currently living children, and identified as Christian (5) or Protestant (4). | Purposive sampling through medical centres, medical staff, and Church pastors. Data were collected using unstructured individual interviews, and analysed using a phenomenological approach with assistance of the NVIvo software package. | All men experienced the loss as deeply significant and devastating. Men reported overwhelming emotions including sadness, crying, frustration, devastation, disappointment, and arguing with God. Many felt angry and helpless that their wives had to ensure pain and suffering. Many compartmentalised their feelings to take control of practical responsibilities and support their spouse. | Fathers used a variety of strategies to manage their emotions, including sport, returning to work, and creativity to express their grief. Validating the loss of the child and men’s sense of fatherhood was important. For many, their faith provided them with the strength to meet the difficulty of their loss. Sensitivity to the needs for privacy, being there, and meeting material needs was helpful and encouraging. |
| Ekelin et al. (2008) | To conceptualize women’s and their partners’ experiences and ways of handling the situation before, during, and after second trimester ultrasound examination with the diagnosis of a nonviable fetus. | Nine Swedish women and six male partners were interviewed within a year of receiving the nonviable diagnosis. For four couples, this was their first baby; one had experienced a miscarriage previously. | Participants were recruited through the ultrasound department at a Swedish regional hospital. Data were collected using in-depth interviews with the couples together; grounded theory was used to analyse the results. | The men felt their roles should be supportive, even though they felt as sad and out of control as the women. Men do not necessarily “move on” more quickly – reactions are individual, and men’s grief may be delayed due to the initial supporter role. | No data pertained to predictors of men’s grief specifically. However, for parents generally, they were affected positively when they were taken care of with empathy. Some discussed concentrating on the children they already had; additional grief was related to work, time of year, and other life circumstances. |
| Fisher (2002) | To describe and explore the lived experience and meaning of miscarriage to the father. | Nine fathers who had experienced miscarriage prior to 20 weeks gestation from the U.S. All were married and Caucasian, and aged between 32 and 49 years. Time since the loss ranged from one week to 10 years. Eight had living children, for one the miscarriage was his first baby. | Convenience sampling occurred through grief support groups, newspaper advertisements and religious newsletters. Each father participated in two semi-structured interviews, data was analysed using phenomenology. | Eight of nine fathers experienced a bereavement response. Miscarriage affected each father, although each had a unique perception: some were devastated, others less affected. The fathers’ need to “do something” was often at the cost of their own feelings. Denial was a coping mechanism for three fathers. Many tried to frame the miscarriage positively to come to terms with it. Anxieties continued into subsequent pregnancies. | The need to offer comfort and support to the mother was expressed by five fathers. A reason for the loss reduced blame and helped fathers to cope. Having people available to talk to about the miscarriage helped fathers to mourn their loss. Al fathers described their relationships with their spouses as loving and supportive, which may have lessened their grief responses. Two fathers saw their baby’s remains (20 weeks gestation), and felt this greatly influenced their grief response. |
| Hamama-Raz et al. (2010) | To examine and understand the meaning ascribed by religious couples, both together and separately, to spontaneous abortion and how this meaning was manifested in their couple relationship. | Five couples from Israel, who had experienced spontaneous abortion (miscarriage) up to the 20^th^ week of pregnancy. Three couples identified as Haredi, and two as Dati-Leumi. All were aged between 26 and 35 years; time since loss ranged between two months and two years. | Recruitment occurred through snowballing within the Israeli religious sector. Data were collected using in-depth interviews with men and women separately, and were analysed using content analysis. | Men reported repressing their painful emotions and resorting to rationalization to create a coping partnership. The initial reaction for men was one of helplessness, however they actively worked to overcome their own sense of shock to support their wives. | The husbands did not express any relationship with the fetus. They preferred to view the fetus as tissue and therefore felt no pain or meaningful loss. Men coped with the loss through logical explanation and rationalization (i.e., “it must mean something was wrong with the fetus”). Religion helped the couple to cope. Men reported a consistent role as comforter throughout the entire process, focussing on strengthening the couple relationship and sense of togetherness. |
| Jones-Peeples (2014) | To understand male partners’ perceptions regarding their experience of perinatal loss as well as the range of psychological and emotional consequences of perinatal loss on men. | Five committed, heterosexual couples from the U.S. participated. All were married and identified as Christian. Males ranged in age from 31 to 41 years, all identified as Caucasian. Couples has experienced two miscarriages (nine and 11 weeks gestation), and four stillbirths (one loss of twins; 20 through to 27 weeks gestation). | Recruitment occurred through internet and social media advertisements, and snowball sampling. Data were collected using in-depth semi-structured interviews with the male partner. The type of analysis used was grounded theory (assisted with the use of NVivo). | Regardless of the length of pregnancy, all men experienced the loss as a significant life event. Initial reactions to the loss included intense shock, disbelief, overwhelming emotions and reactions including crying, confusion and disappointment. All expressed feelings of fear, anxiety, helplessness and anger that their wives had to ensure great amounts of physical pain. The men noticed changes in their behaviours, including increased sadness and crying, decrease in social and physical activities, difficulty sleeping and physical exhaustion. | All of the men described supporting their wives throughout the process, and many expressed feeling like they “needed to be strong” and even suppress their own feelings to support their wives. While men described reviewing less support from medical personnel, family and friends, many wanted their wives to be more supported due to their physical and emotional trauma. Men used a variety of strategies to manage their own grief, including work, exercise, and staying busy, and their faith/church. Finding ways to remember and validate the life of their child helped men to grieve and move forward. |
| Kavanaugh & Hershberger (2005) | To examine the experience of low-income, African American parents surrounding perinatal loss and to describe how other life stressors influenced the parents’ responses and caring needs. | 17 mothers and six of their male partners who had experienced perinatal loss participated. 11 couples had experienced stillbirth between 17 and 37 weeks gestation, and six had experienced neonatal death between one and 28 days after birth. Six couples had experienced prior perinatal loss. Fathers ranged in age from 20-34 years. | Participants were recruited through a newspaper advertisement, along with referrals from hospital staff. Data were collected using two individual interviews with each parent; follow-ups occurred between two and five weeks following the first. Data were analysed using a descriptive phenomenological approach. | Parents experienced intense feelings; fathers emphasised that they also hurt and experienced a loss of control. Men reported keeping their emotions under control for fear of further upsetting the mother. Although they wanted to support their partners, they were unsure how to do so. | All but one father saw their baby; most fathers were very reluctant to hold their baby but did so with encouragement from nurses or family members. This time with the baby was cherished by parents. Fathers, more so than mothers, found it helpful to keep busy, to “move forward,” and to think positively about the future. |
| Kelley & Trinidad (2012) | To examine parents’ and physicians’ experiences and beliefs surrounding stillbirth within the context of the clinical encounter. | 22 U.S. bereaved parents to stillbirth and obstetric/gynaecologic health professionals participated: two of which were fathers. No further demographic data was collected. | Participants were identified and initially recruited through parent hospital guild groups, followed by snowball sampling within these groups. Data were collected using three semi-structured focus groups, and analysed using thematic discourse analysis. | The grief experience following stillbirth was ambiguous; one father described his grieving process as uncertain and changing over time. Both fathers described feeling sadness or depression, but supporting their wives was their primary focus. | A sense of constrained grieving was caused by social discomfort and taboo which extended to husbands and grandparents, who were not expected to grieve the loss of a stillborn baby beyond feeling some transient disappointment or sadness for their wife or daughter. |
| Lukas (1999) | To reveal the characteristics and dimensions of the process of experiencing paternal grief, mourning, and adaptation following perinatal loss. | 15 U.S. men who had experienced perinatal loss, ranging in age from 28-52 years. Gestational age of their babies at time of loss was 20-42 weeks, and time since loss ranged from 5-98 months. All men were married and employed in full-time work. | Recruitment occurred through perinatal loss support groups. Data were collected using semi-structured individual interviews and administration of the Perinatal Grief Scale. | Each of the fathers recognised and acknowledged their loss as one of a baby. Common grief responses included alienation, life change, frustration, sadness, shock and helplessness. Unique elements of grief included disappointment, ritual, disenfranchisement, attachment and masculinity. Fathers experienced disenfranchised grief in the sense that the attention and focus was on their female partner, both in the healthcare system and in relation to family/friends/community. | The most common expressions positively affecting or facilitating grief were reported to be support group participation, rituals, positive healthcare experiences, returning to work, social support, and family. Most fathers expressed gratitude for support groups, however some felt they impeded grief adaptation due to the “female-centred” nature. Positive experiences with the hospital led to both increased support group participation and improved grief outcome. Returning to work gave fathers a break from grief. Only five felt that family members were a positive support; others were disappointed with the support they received from them. |
| McCreight (2004) | To examine the impact of miscarriage and stillbirth on male partners in Northern Ireland. | 14 men who attended self-help groups for pregnancy loss. Three men had experienced a miscarriage, six a stillbirth, and five both miscarriage and stillbirth. Gestational ages at the time of loss ranged from seven to 41 weeks; two men had no living children. Men’s ages ranged from 21 to 43; time since loss ranged from two months – 20 years. | Participants were recruited through local pregnancy loss support groups. Data were collected using semi-structured, in-depth interviews. A narrative approach to analysis was taken using content analysis, assisted by the use of NVivo. | Men in this study grieved deeply following their loss. Irrespective of time lapse since the bereavement, all appeared to have suffered. Many questioned their identities as fathers. All of the fathers in the study expressed a need to put their own grief and emotional needs aside in order to support and comfort their partner. | Most of the men saw an ultrasound scan of their unborn baby; these fathers reported developing an awareness of the baby as a real living person. Not all men had access to a birth certificate for their babies; this prevented them from having their experience of death validated. Several men reported being marginalised by hospital staff, despite the fact that they had onerous responsibilities at the time. All fathers indicated that they frequently felt their loss had been devalued by the wider community. Self-help groups also afforded to the men an opportunity to grieve through rituals and remembrance services. |
| Meaney et al. (2017) | To explore the experiences of those who have experienced miscarriage, focusing on men’s and women’s accounts of miscarriage. | 16 Irish parents bereaved by miscarriage, six of which were men. Gestational age at the time of loss ranged between six and 16 weeks, and time since loss ranged from 18-96 months. The men had a history of two to seven previous miscarriages, and the number of living children ranged from three to none. | Participants were initially recruited through women’s previous participation in a prospective cohort study on miscarriage, then through snowballing techniques. Data were collected using semi-structured individual interviews, and analysed using interpretive phenomenological analysis. | Men reiterated that although they did not experience the miscarriage physically, they were affected emotionally and did go through a grieving process. Men felt that their primary role was to support their partners through the loss and, at times reluctantly, while planning subsequent pregnancies. | Rituals to acknowledge the loss were extremely important to all participants. Keeping busy helped participants cope with their loss; this was particularly evident in the participants who already had children. Men in this study indicated that they were less likely to openly discuss the miscarriage unless prompted by another person with a similar experience. |
| Murphy (1998) | To describe the experience of early miscarriage from a male perspective using a phenomenological approach. | Participants were five men who had experienced a miscarriage prior to 24 weeks gestation. All losses had occurred more than two years prior to the interview. | Purposive, snowball sampling was used to recruit participants. Data were collected using unstructured individual interviews, and analysed using a phenomenological approach. | Miscarriage was a sudden, unexpected event. Initial feelings included shock, disbelief, upset and helplessness. All men felt concern for their female partners. There was a perception that early miscarriage provoked a more intense reaction for their partners than for themselves. Men described an expectation that they should be stronger and tougher in order to support their partner and have no need to grieve or share their feelings. | Participants indicated that the intensity of men’s grieving may be related to the extent to which the foetus seemed ‘real’ to them. A common coping strategy the men used was to ignore things, try to forget the miscarriage and carry on with life as normal. All participants also commented that they felt very alone in trying to cope. Most support came from friends, family and their partner, but not from health professionals. |
| Obst (2017) | To explore Australian men’s experiences of support following a female partner’s pregnancy loss. | Participants were eight Australian men who had experienced a pregnancy loss between six months and five years ago, and seven pregnancy loss service providers. Men were aged between 33 and 45 years, all we in a de facto or married relationship. Losses occurred between 20 and 31 weeks of gestation, and included several “early miscarriages” prior to 20 weeks. | Recruitment occurred through local pregnancy loss support organisations and social media advertisements. Data were collected using semi-structured individual interviews and analysed using thematic analysis. | Men described varied experiences with grief and subsequent support needs. Grief was not related to gestational age, but rather an individual experience. Many men described how a lack of recognition for their grief made the grieving process even harder, as they felt as though they had to suppress their feelings. Participants downplayed their grief experiences in relation to that experienced by women, given that their female partners had endured the physical component of the pregnancy and loss. | Many men described practical responsibilities as impacting their grief experience, including: caring for other children, work, and “trying to keep everything normal at home”. The most helpful health professionals were those who were patient with men and gave them space to process their loss and grief. The majority of men recounted positive experiences with helpful family members or friends, especially in the earliest stages following the loss. All of the men mentioned a feeling of being a ‘supporter’ to their female partner and children. Stigma around men’s help-seeking and societal expectations for how men should behave in relation to grief and loss served as a barrier to accessing support for grief. |
| O’Leary & Thorwick (2006) | To present information about the father’s perspective during the experience of a pregnancy following perinatal loss. | Participants were 10 U.S. fathers whose female partners were currently pregnant subsequent to a perinatal loss. Ages ranged between 28 and 59 years. Five fathers had one living child each, one father had two. All losses occurred within one year prior to participation. | Recruitment occurred through friends of former parents who had been involved in a pregnancy loss support group, advertisement in a bereavement newsletter, and staff referrals at a perinatal centre. Data were collected using individual interviews, and analysed using descriptive phenomenology. | Pregnancy (and loss) was regarded as a women’s experience, and fathers felt ignored. Fathers described how “life goes on” in spite of the need to grieve. They were exhausted, physically and emotionally, but when asked how they managed, a common response was “I keep myself busy”. Fathers made an effort to appear strong, but their overt behaviour contradicted their inner state of stress and vulnerability. Protecting their partner can impede fathers from dealing with their own feelings. | Lack of recognition was identified as a societal issue by six fathers; they described feeling overlooked and wanted to be seen as more than a “support person”. Many had Some had obligations of older children and manifold household tasks if their partner was on bed rest. They were not given time off from work like their female partners/wives. Societal pressure for men to be “the strong one” created a tremendous burden and was a barrier for securing much needed support. |
| Samuelsson et al. (2001) | To describe how fathers experienced losing a child as a result of intrauterine death. | Participants were 11 Swedish fathers who had experienced stillbirth between 32 and 42 weeks gestation. Fathers’ ages ranged between 31 and 46 years, and time since loss ranged between five and 27 months. Eight were married to, and three cohabiting with, the mother of their stillborn child. For five fathers the stillborn child was their first child. | Participants were recruited through a Swedish hospital (not specified). All fathers whose offspring died during weeks 29 to 42 between 1997 and 1998 were invited to participate. Data were collected using individual interviews, and analysed using phenomenology. | Following the loss, fathers wept and felt severe grief, meaninglessness, abandonment, emptiness, guilt, and fear of the abnormal. They didn’t think so much about themselves but tried first of all to protect their partner. They tried to take care of practical matters themselves, however grief was exhausting and made it difficult to cope with these tasks. At times, they felt left behind, outside of it all, confused, and submerged in a totally female-dominated world. There was a need to be left alone, but at the same time they needed help and support. | The primary elements in fathers coming to grips with their grief were tokens of remembrance and support from the obstetric staff and hospital chaplains. Having had children previously was thought of as source of strength and an asset that facilitated their everyday lives. The most valuable help in everyday life was the good relationship with their partner. Most also received good support from relatives, friends, and fellow workers, although they felt that their partner received more. They sought recognition as mourning fathers from caregivers and significant others. |
| Tennenbaum (2008) | To gain insight and a more comprehensive portrayal of the range of psychological and emotional consequences of recurrent miscarriage on couples. | 15 U.S. couples who had experienced the loss of three or more consecutive pregnancies prior to the 20^th^ week of gestation. Age of the couples ranged from 23 to 49 years; 11 identified as White/Caucasian, three as Hispanic/Latino and one as Black. Couples had experienced between three and 10 miscarriages, and time since the last ranged from one to 18 months. | Recruitment occurred through internet advertisements on miscarriage-related websites, invitations to couples known by the researchers, and snowball sampling. Data were collected using in-depth, semi-structured interviews over the phone, and were analysed using grounded theory. | All men expressed grief, although specific manifestations differed. All men expressed sorrow and/or deep disappointment during the miscarriages. Most of the men reported a real sense of loss and great difficulty coping – they cried both alone and with their wives. With each miscarriage, men lost more and more hope that they would ever have a biological child. Several husbands reported difficulty meeting expectations of their roles as husband, support, caretaker, as well as family and medical liaison. They reported a need to protect their wives, even if it was at their own expense. | For most of the men, attending the ultrasound or seeing images was a significant experience that often compounded their grief – it made the pregnancy more of a reality and increased attachment. A lack of answers significantly affected all the husbands. Men became angry when they felt that their grief was ignored; they felt that they needed to be strong and not show emotion. Sharing their loss with family, friends or their church community was helpful. A few men reported friction in their marriage due to different coping strategies, however all believed the loss brought them closer together. |
| Wagner et al. (2018) | To examine the lived experiences of fathers who have experienced miscarriage. | 11 U.S. fathers who had experienced a miscarriage before 24 weeks gestation. Each was currently married to the mother of the lost pregnancy. Several had experienced multiple miscarriages, nine had since experienced a successful pregnancy. Nine identified as Caucasian, two as Black. | Participants were recruited using convenience sampling of personal contacts and social media, followed by snowball sampling. Data were collected using face-to-face semi-structured interviews, and analysed using phenomenology. | Participants perceived themselves as fathers, and reported taking their responsibilities as one “very seriously”. An expressed need to provide, protect and nurture led the fathers to question whether they could have done more to prevent the loss. Fathers recalled being focused on the health and wellbeing of their partner and care of their families; these responsibilities needed to be balanced with their own grief. Fathers described the need to be strong for the mother, which impacted their ability to experience grief. | Belief that the miscarriage was the loss of a person, rather than the loss of a pregnancy, seemed to make processing more difficult. Support from others (family and friends) who had also experienced miscarriage was particularly meaningful. Practical support was helpful (e.g., cooking meals, time off from work and extensions on projects, gift baskets). Fathers often experienced disenfranchisement indirectly by not being recognized as a part of the process: although they believed the mother should be the primary focus, they often felt ignored by others. |
| Wagner et al. (1998) | To explore with fathers their perinatal death experiences. | 11 fathers who had experienced perinatal death (conception to 28 days after birth) in the U.S. Fathers ranged in age from 28 to 38 years, all identified as non-Hispanic White. Seven losses occurred before birth, and four experienced a neonatal death. Time since loss ranged from six months to five years. | Participants were recruited through a community support group called “Hoping”. The facilitator of the group referred 11 participants from the group to participate. Data were collected using structured telephone interviews, including numerically rated responses and open-ended questions. | Fathers were asked to rate their grief at the time of perinatal death from 1 (no grief) to 10 (severe grief). The mean response was 8.5, however higher scores were reported for second trimester and neonatal losses. Those who had experienced first trimester loss had a mean score of zero (no grief) at the time of interview. Only one father denied having cried in response to his loss (first trimester). | Only half of the fathers felt they were supported during the experience; nine fathers reported their partner as their main source of support in grief. 72% felt there was a difference between support for them and their partner from family, friends and community; with the wife receiving more support and the majority of fathers feeling “overlooked”. In general, grief decreased with time since death. However, the majority of fathers expected to grieve throughout their lifetime. |
| Weaver-Hightower (2012) | To consider the experience of the author’s own daughter’s stillbirth, exploring grief, tactile contact with death, and how these demonstrate the strictures and ruptures of masculinity in Western cultures. | Autoethnography of one father’s experience with the stillbirth of his daughter at 38 weeks’ gestation. | Authoethnographic methods were used, including interviewing others, using self-artefact and photo elicitation, reflective memoing, participant observation in bereavement support groups, and creating art and scrapbooks. | Grief came in “flickers and fragments”, the author felt that the loss ruined everything, and challenged his religious faith. He felt helpless for his wife’s suffering, and wished to fix it. Additional roles (such as informing people of the loss) fell to the father. He felt as though he had to hide his grief and work to suppress any form of emotional expression. | The author developed a strong attachment to his daughter throughout pregnancy. He recognised that his grief was highly social, and regulated according to gender and other social and cultural inequalities. Mementoes performed a “critical role”, along with instrumental coping strategies (e.g., woodworking), which served as a form of therapy which was not talking. Returning to work was difficult, and he felt that he could not take off the time to mourn. |

Quantitative Studies

| Authors (Year) | Study Aims (objectives; focus) | Participants/Setting (*N* of men; loss type; country; time since loss) | Method/Design (recruitment; data collection; analysis) | Measure of Grief (grief measure used) | Predictors/Outcomes (key predictors and/or grief outcomes) |
| --- | --- | --- | --- | --- | --- |
| Alderman et al. (1998) | To explore the psychological impact of grief and stress in couples who experienced a miscarriage. Investigated differences between the couple in how they grieve and experience the stress of miscarriage | 19 Caucasian, married couples; 10 experienced a miscarriage in their first pregnancy; loss occurred within two years of study; fathers were an average of 37 years; undertaken in the U.S. | Recruitment strategy not specified; data collected via a questionnaire using validated psychological instruments. | The Grief Experience Inventory-Loss Version Impact of Event Scale (IES) | Men’s overall responses differed significantly to that of women’s; men had elevated scores on the avoidance subscale of the IES. |
| Barr (2004) | To explore the relationship of guilt- and shame-proneness to grief in bereaved parents 1 month (‘early’) and 13 months (‘late’) after a stillbirth or neonatal death. | 86 mothers and 72 fathers bereaved by stillbirth (≥20 weeks gestation) or neonatal death (≤28 days after birth) in Australia; mean age of men was 34.4 years; ethnicity was primarily English-Australian (75%); majority of parents were married (70%) or cohabiting (23%). | Parents who had experienced a stillbirth in four (of six) high-risk obstetric hospitals in Sydney were sent an invitation letter 2-3 weeks after the loss; semi-structured interviews and a questionnaire were completed in parents’ homes or over the phone. | Perinatal Grief Scale-33  **Other measures:** Test of Self-Conscious Affect-2 (TOSCA-2) Personal Feelings Questionnaire (PFQ) Interpersonal Guilt Questionnaire (IGQ-67) | At one month, women reported more intense grief, but at 13 months there were no sex differences in grief. Shame and guilt together explained 27% of the variance of early grief (one month after the loss) in men. For late grief (13 months after the loss), shame and guilt together accounted for 63% of the variance in men. |
| Barr (2006) | To explore the relationship between parental grief following perinatal bereavement and subsequent pregnancy, according to the particular facets of grief and pregnancy state being considered. | Participants were 63 heterosexual Australian couples who had been bereaved by stillbirth (prior to 20 weeks gestation; n = 31) or neonatal death (death within 28 days of birth; n = 32). Participants were aged between 19 and 50 years of age. The majority identified as English-Australian (76%), European-Australian (10%), or Asian-Australian (8%). | Recruitment method is not specified; 44% of eligible parents from participating hospitals agreed to participate. Data was collected using two semi-structured interviews with parents, approximately one month and 13 months after the loss. Parents also completed a psychometric measure of grief. Results were analysed using repeated-measures ANOVAs. | Perinatal Grief Scale-33 | The presence of living children before loss was not significantly correlated with Perinatal Grief scores at one or 13 months. A significant main effect for the Active Grief subscale of the PGS was found in men. The pregnancy status interaction was not significant for men, indicating that subsequent pregnancy status did not have an effect on grief. |
| Barr (2012) | To examine the intrapersonal (actor) and interpersonal (partner) relationships of personality proneness to negative self-conscious emotion (shame and guilt) to grief in couples 13 months after a perinatal death. | Participants were 63 heterosexual Australian couples who had been bereaved by stillbirth (prior to 20 weeks gestation; n = 31) or neonatal death (death within 28 days of birth; n = 32). The majority identified as English-Australian (76%), European-Australian (10%), or Asian-Australian (8%). | A letter was posted to eligible parents 2–3 weeks after the perinatal death, and 1 week later they were contacted by telephone to ascertain their willingness to participate in the study. Data was collected using self-reported questionnaires containing validated psychometric measures, and analysed using regressions and paired samples t-tests | Perinatal Grief Scale-33  **Other measures:** Test of Self-Conscious Affect-2 (TOSCA-2) Personal Feelings Questionnaire (PFQ) Interpersonal Guilt Questionnaire (IGQ-67) | Personality proneness to shame and proneness to guilt were shown to predict grief intensity in parents bereaved by stillbirth or neonatal death, and the predictions were invariably stronger in men compared with women. Analysis showed that negative self-conscious emotion had an intrapersonal (actor) relationship with grief in men. |
| Conway & Russell (2000) | To investigate the grief response of both the woman and her partner to miscarriage and to ascertain if support received was adequate and appropriate to their needs | 39 women and 32 male partners who had experienced miscarriage in Australia. Losses occurred between 5 and 16 weeks gestation, 12 males had surviving children prior to the loss. | Purposive sampling through four major Sydney obstetric hospitals and one district hospital. Data were collected using two round of questionnaires, the follow-up 2-4 months later. | Perinatal Grief Scale | Age, length of relationship with partner, education, previous children and socio-economic status were not related to grief scores. Initial reactions were very sad (53%) and sad (34%); 75% of men said their partner’s reaction affected them. Feelings of loss remained 2-4 months later for 63%; 32% still thought about it daily. Partners scored significantly higher than the women on the three PGS subscales and overall. 94% reported they were able to talk to their partner about the loss; 73% of partners rated support from relatives and 71% from friends as helpful. Only 18% of men were asked by hospital personnel how they were coping with the miscarriage – 46% said they would have liked to have been. |
| Cope et al. (2015) | To examine the psychological impact, specifically symptoms of grief, post-traumatic stress and depression, in women and men who either terminated or continued a pregnancy following prenatal diagnosis of a lethal fetal defect. | 158 women and 109 men who had lost a pregnancy or baby due to anencephaly. Study was based in the U.S. but also included 15 participants from UK, Canada and Australia. Ethnic backgrounds were primarily non-Hispanic Caucasians (90%), with men aged between 20 and 42 years at the time of loss. Time since loss varied between one month and 32 years. | Participants were purposively sampled through their participation in a prior study on Neural Tube Defects, and through social media advertisements for the present study. Data collection occurred through survey questionnaires using validated measures and project-specific questions. | Perinatal Grief Scale-33 Impact of Events Scale – Revised | Men’s scores on the PGS ranged from 37 to 120 with 11% scoring in the pathogenic range for grief. Time since pregnancy was significantly associated with scores on the PGS and IES; those with more recent losses scoring higher. Pregnancy continuation was associated with higher scores on the difficulty coping subscale, whereas termination in the second trimester was associated with higher active grief. For participants who continued the pregnancy, there were no significant differences in psychological outcome between those who had a stillborn or live-born baby (neonatal death). |
| Franche (2001) | To determine if the psychologic constructs of self-criticism and marital adjustment, considered jointly with obstetric and demographic factors, are significant predictors of grief during a pregnancy after a miscarriage or perinatal death | 60 Canadian women between the 10^th^ and 19^th^ week of gestation, and 50 of their partners. Men were aged between 24 and 46 years. Gestational age at the time of prior loss ranged between four and 42 weeks; three had neonatal losses within four days after birth. Time since the loss ranged from four to 48 months. | Purposive sampling through obstetric clinics and hospital staff in a large Canadian University Hospital. Participants completed a battery of self-report questionnaires containing validating psychometric scales. | Perinatal Grief Sale–Short Form  **Other measures:**  Depressive Experiences Questionnaire—Self-criticism subscale Abbreviated Dyadic Adjustment Scale | In men, only gestational age at time of loss and time between loss and conception were significant predictors of grief levels. Age, number of previous losses, and number of living children, when considered in conjunction with psychologic variables, were not significantly associated with grief levels. Gestational age at time of loss was a significant predictor of active grief for men. |
| Franche & Bulow (1999) | To examine the impact of a subsequent pregnancy on emotional adjustment associated with a previous perinatal loss and on components of parental grief. | 25 Canadian women and 24 partners expecting a baby for the first time since a prior perinatal loss, and 25 women and 18 partners who were not expecting or had a child since a prior loss. Fathers were aged between 26 and 51 years; losses occurred between 10 and 42 weeks gestation. Time since loss varied from 1 month to 31.5 months. | Purposive sampling through obstetric clinics in a large Canadian University Hospital. Participants completed a battery of questionnaires containing validated psychometric scales. | Perinatal Grief Scale–Short Form  **Other measures:** Beck Depression Inventory State-Trait Anxiety Inventory Abbreviated Dyadic Adjustment Scale | For the fathers, two MANCOVAS were performed examining group differences in levels of grief and emotional adjustment. No significant group effects in emotional adjustment or intensity of grief were found between the “pregnant loss” and “loss” groups, indicating that subsequent pregnancy status did not impact upon men’s emotional and grief outcomes. |
| Huffman et al. (2015) | To understand the effect of gender, age, mental health history, and reproductive factors on the appraisal of miscarriage in couples. | 341 couples who had experienced a miscarriage in the U.S. participated. 85% of participants were White; 4.7% black; 5.6% Asian/pacific Islander; 3.2% Hispanic; and 0.6% Native American. | Participants were part of a larger study called the Couples Miscarriage Healing Project, a randomised controlled clinical trial. Data for this study were responses to a validated psychometric scale collected at baseline. | Revised Impact of Miscarriage Scale | Women scored significantly higher than men on all measures. Younger age and more advanced gestational age at time of loss were associated with higher scores on the isolation/guilt and loss of baby subscales. Men who had living children had higher scores on the devastating event and loss of baby subscales. Mental health treatment, infertility, and miscarriage history did not affect the impact of miscarriage in men. |
| Jansson et al. (2017) | To compare Swedish and American couples’ experience of miscarriage by use of the Revised Impact of Miscarriage Scale. | Participants were Swedish (*n* = 70) and American (*n* = 70) couples who had experienced miscarriage up to 21 weeks gestation. The median week of miscarriage was 10.2 and 10.0 weeks for the Swedish and American couples, respectively. Couples were matched according to age, number of miscarriages and previous children. | Swedish couples were recruited through a gynaecology emergency clinic at Uppsala University Hospital. American participants were part of a larger miscarriage project. Data were responses to a validated self-report psychometric scale. | Revised Impact of Miscarriage Scale | The American men scored significantly higher on the factor ‘Loss of baby’ than the Swedish men. There was no difference between the scores on Isolation/Guilt or Devastating Event. |
| Johnson & Puddifoot (1998) | To explore the role of visual imagery in mediating the male grief reaction to a partner’s miscarriage. | Participants were 158 British men who had experienced a miscarriage prior to the 24^th^ week of gestation. Miscarriages occurred between the 6^th^ and 24^th^ week of pregnancy. Time since miscarriage was within 11 weeks. All participants were in ‘stable’ relationships. 38% of couples had no previous children; 29.7% had one and 32.3% had two or more. Half of the couples has experienced miscarriage previously. | Participants were purposively sampled through gynaecological wards of general hospitals and several large GP surgeries. Data were collected via a self-report questionnaire using validated psychometrics scales. | Perinatal Grief Scale  **Other measures:** A modified form of the Vividness of Visual Imagery Questionnaire (baby focus) | Vivid imagers exceeded the scores on all elements of the PGS in comparison to mid-range and non-vivid imagers. Scores on the difficulty and despair subscales specifically were exceedingly high, comparable to those found for women who have experienced miscarriage. Vividness of imagery had a highly significant effect on PGS score overall. Low scores on the active grief subscale suggest that men are likely to be less demonstrative in their expression of grief. |
| Khan et al. (2004) | Aims were: (1) to assess the emotional responses of men to early pregnancy loss; (2) to establish if sufficient support services are provided to these men; (3) to make recommendations to improve the quality of these support services, if necessary. | Nine men who were attending an early pregnancy loss clinic in Dublin with their female partners participated. Men were aged between 20 and 39 years. All were Caucasian, and had experienced a miscarriage at less than 20 weeks gestation. | Data were collected using a study-specific questionnaire comprising of open and closed questions. Data were presented in the form of percentages of responses to questions. | No validated measure of grief. | Men described a range of feelings in response to the miscarriage, including uncertainty, sadness, blank, confusion, and anger. None were satisfied with the level of support and information received at the time of the miscarriage; they felt all support services for their partner were satisfactory but they were excluded. Some found comfort by crying, support from family/friends, or discussion with their partner. |
| Michon et al. (2003) | To evaluate the intensity of grief experienced by parents who have lost a child in the perinatal period (stillbirth, premature baby, term baby less than one month) and parents who have lost a child after the perinatal period (one month to 18 years). | All parents who had lost a child in Estrie, Quebec between Jan 1997 and Dec 1999 were contacted to participate in the study. The questionnaire was sent to 85 families and 71 parents returned them completed (32 of which were perinatal loss parents). Gestational age for the losses ranged between 24 and 41 weeks, and time since loss ranged between 36±9 months. 79% of parents identified as Catholic. | Data were collected using a self-reported questionnaire containing | Texas Revised Inventory of Grief [TRIG-F] | Fathers’ grief was less than that of mothers. No correlations between the TRIG-F scores and gestational age were observed. Early in their grief, fathers reported finding it hard to work after the loss. Long-term, fathers still felt the need to cry for their baby, and felt anxious and upset at anniversaries and events. Long-term grief scores averaged higher than early grief scores (31±12 versus 17±9, respectively). |
| Puddifoot & Johnson (1999) | To investigate male’s grief responses to miscarriage (as measured by the Perinatal Grief Scale), and contributing characteristics. | 323 men from the UK who had experienced a miscarriage prior to the 24^th^ week of pregnancy. Participants’ ages ranged between 17 and 56 years, and approximately half had suffered a miscarriage previous to the last. Time since the last miscarriage was within 2-8 weeks prior to data collection. | Recruitment strategy is not specified – results were from two separate but methodologically identical studies. Data were collected using self-report questionnaires. | Perinatal Grief Scale | Analysis revealed high levels of grief on the PGS, but with considerable variation. Taken together, the high mean scores and the relatively large dispersion of scores indicate both a diversity of response and a generally raised level of grief. Grief increased gradually with duration of pregnancy before miscarriage. The level of grief of those who had seen an ultrasound scan was also considerably higher. |
| Rich (2000) | To determine the impact of post-pregnancy loss services on grief outcome in both bereaved mothers and their male partners. | 249 bereaved mothers and 114 male partners from the United States and Canada returned eligible completed research packs. Couples experienced between one and 12 previous losses, between three and 42 weeks gestation. Time since loss ranged from two to 60 months. 95% of participants identified as Caucasian; 68% as Christian, Catholic or Protestant, and 33 as Jewish. | Recruitment occurred through a variety of means, including: online and in-person support groups, parent newsletters, nurse/social work referrals, and church bulletins. Data were collected using self-reported questionnaires which were mailed to parents. Data were analysed using a stepwise multiple regression analysis. | Perinatal Grief Scale – Short Form | Following the loss, 96.4% of fathers reported talking with family, and 91.2% with friends. The majority of fathers reported meeting with a doctor to review reasons for the loss (82.5%), or plan for a future pregnancy (75%). In the final regression model for fathers, length of pregnancy, talking with friends, and timing of talking with family were significant predictors of grief; accounting for 15.5% of the total variance in PGS scores. |
| Serrano & Lima (2006) | To describe the consequences of recurrent pregnancy loss for the couple’s relationship and explore gender differences in attitudes and grief intensity. | 30 couples from Lisbon who had experienced recurrent miscarriage up to 24 weeks of gestation. Men were aged between 24 and 51 years; 76.7% identified as Catholic and 16.7% as atheist. 28 couples were Caucasian and two were Black. The majority of couples (76.7%) had experienced three miscarriages, 20% had experienced four, and one couple had six. Time since the most recent loss ranged from three months to one year. | Participants were recruited at the Recurrent Miscarriage Clinic at Maternity Dr Alfredo da Costa in Lisbon. Data were collected using self-reported questionnaires which couples completed in separate areas of the waiting room prior to their appointment at the clinic. | Perinatal Grief Scale Impact of Events Scale  **Other measures:** Intimate Relationships Scale Partnership Questionnaire | Both the PGS and IES revealed high levels of grief and stress associated with recurrent miscarriage for men. Duration of relationship, ethnic background, number and duration of gestations, and time since last miscarriage were not significantly associated with PGS or IES scores. Grief was related to the perceived change in sexual relationship only for men; the higher the suffering, the lower the perceived quality. |
| Swanson et al. (2009) | To examine the effects of three couples-focused interventions and a control condition on women and men’s resolution of depression and grief during the first year after miscarriage. | The final sample for analysis consisted of 636 U.S. participants (315 men) who had experienced miscarriage prior to 20 weeks gestation. Gestational age at loss ranged from 2.7 to 20 weeks, however 95% miscarried prior to 16 weeks. The majority of men identified as White (86%), and were an average of 34 years of age. | Participants were recruited from the Washington area via posters, print and media advertisements, and pamphlets in healthcare facilities. A randomised control trial, couples were randomly assigned to nurse care (NC), self-care (SC), combined care (CC) or a control (no treatment) intervention. Data were collected using mailed surveys at approximately one (baseline), three, give and 13 months after miscarriage. | Two subscales from the Miscarriage Grief Inventory were used: Pure Grief and Grief-Related Emotions. | Men and women responded differently to miscarriage. Both CC (a combination of nurse and self-care interventions) and NC (nurse care intervention only) offered strong evidence of hastening men’s resolution of Pure Grief and Grief-Related Emotions above SC (self-care intervention). Men randomised to no treatment also resolved their Grief-Related Emotions faster than men in SC. This study concluded that one nurse counselling session followed up with videos and workbooks may have provided sufficient coaching to help men come to terms with their own transitional responses to miscarriage. |
| Volgsten et al. (2018) | To study the emotional experience, grief and depressive symptoms in women and men, one week and four months after miscarriage. | 103 women and 78 men who had experienced a miscarriage in Sweden prior to 22 weeks of gestation. Men were aged between 25 and 51 years, 96% were married to, or living with, their partner. Just over half (57.7%) had previous children, and 7.7% had experienced infertility issues. | Participants were recruited through a gynaecological clinic after miscarriage. Data were collected using self-reported questionnaires containing validated psychometric measures. | Revised Impact of Miscarriage Scale and the Perinatal Grief Scale | The relative emotional experience of miscarriage did not change significantly from one week to four months in women and men. For the men, all three subscales of the Perinatal Grief Scale were reduced after four months compared to one week. |
| Wilson et al. (2015) | To document parents’ experiences and outcomes in relation to seeing and holding a stillborn baby at a hospital with a specialist perinatal bereavement service. | 26 mothers and 11 male partners who had experienced a stillbirth after 20 weeks gestation in Brisbane, Australia. Median gestational age for all losses was 27 completed weeks. 18 births were singletons, seven were twin births. The majority of participants were Caucasian. | Participants who experienced a stillbirth at the Mater Mother’s Hospital in Brisbane between Sept 2007 and Dec 2008 were invited to participate in the study. Data were collected using mailed self-report questionnaires, and analysed using mixed-effects linear regression models. | Perinatal Grief Scale  **Other measures:** Decisional Regret Scale and Mental Health Inventory | Nine fathers chose to see and hold their baby. One agreed or somewhat agreed that their choice did them ‘a lot of harm’. Fathers who did not see and hold had significantly better mental health in all three post-loss surveys than those who did. There was a significant difference between participants who held and did not hold on the active grief subscale; however due to the small number of fathers, the estimates are imprecise. |

Mixed Methods Study

| Authors (Year) | Study Aims (objectives; focus) | Participants/Setting (*N* of men; loss type; country; time since loss) | Method/Design (recruitment; data collection; analysis) | Measure of Grief (grief measure used) | Results/Outcomes (key predictors and/or outcomes of grief) |
| --- | --- | --- | --- | --- | --- |
| Avelin et al. (2013) | To describe the grief of mothers and fathers and its influence on their relationships after the loss of a stillborn baby. | 55 parents (22 fathers) bereaved by stillbirth (> 22 week’ gestation) from the Stockholm region in Sweden; loss was the first child for 50% of fathers; mean age of men was 33; all parents were married or cohabiting. | All parents who experienced a stillbirth over a one year period at a hospital (86 babies) were given details of the study by a midwife after the stillbirth, before they left hospital (twin deliveries excluded). Data were collected through a mixed methods postal questionnaire at 3 months, 1 year and 2 years after the loss. | The researcher-developed questionnaire comprised multiple choice questions with space for comments, and some open-ended questions covering: demographic data, time before and during pregnancy, the delivery, contact with the stillborn baby, grief reaction, situation for any siblings, partner relationships and intimacy. | **Quantitative findings:** 82% of fathers did not feel they were grieving for their baby in the same way as their partner; however, at one year all had been able to talk to their partner ‘to a great extent’ about the loss. **Qualitative findings:** Men more often expressed their grief through frustration, activities or withdrawal. Others just quickly wanted to work through their grief and move forward with their lives. Relationship difficulties arose due to the expectation for the father to be the supporter and not show his feelings. |
